# Quantification of Distributions of Local Proton Concentrations in Heterogeneous Soft Matter and Non-Anfinsen Biomacromolecules

**DOI:** 10.1021/acs.jpclett.4c00825

**Published:** 2024-05-17

**Authors:** Sergei Kuzin, Dario Stolba, Xiaowen Wu, Victoria N. Syryamina, Samy Boulos, Gunnar Jeschke, Laura Nyström, Maxim Yulikov

**Affiliations:** † Department of Chemistry and Applied Biosciences, ETH Zurich, Vladimir-Prelog-Weg 2, 8093 Zurich, Switzerland; ‡ Department of Health Sciences and Technology, ETH Zurich, Schmelzbergstrasse 9, 8092 Zurich, Switzerland; § Max Planck Institute of Colloids and Interfaces, 14476 Potsdam, Germany; ∥ Voevodsky Institute of Chemical Kinetics and Combustion, Novosibirsk 630090, Russia

## Abstract

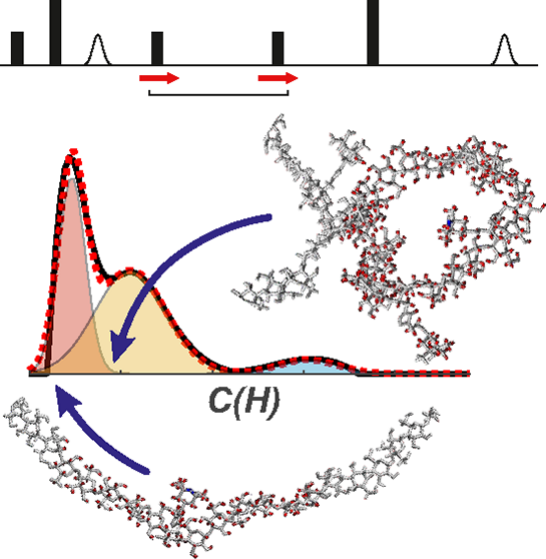

A new method to quantitatively analyze heterogeneous distributions of local proton densities around paramagnetic centers in unstructured and weakly structured biomacromolecules and soft matter is introduced, and its feasibility is demonstrated on aqueous solutions of stochastically spin-labeled polysaccharides. This method is based on the pulse EPR experiment ih-RIDME (intermolecular hyperfine relaxation-induced dipolar modulation enhancement). Global analysis of a series of RIDME traces allows for a mathematically stable transformation of the time-domain data to the distribution of local proton concentrations. Two pulse sequences are proposed and tested, which combine the ih-RIDME block and the double-electron–electron resonance (DEER) experiment. Such experiments can be potentially used to correlate the local proton concentration with the macromolecular chain conformation. We anticipate an application of this approach in studies of intrinsically disordered proteins, biomolecular aggregates, and biomolecular condensates.

In the past few decades, our view of the differences and similarities between biomacromolecules and synthetic polymers in solution has been gradually changing. In the middle of 20th century, after Anfinsen’s works^1^ on the one side and the development of the polymer solutions theory^2−4^ on the other side, proteins were considered as structured macromolecules assuming a single conformation encoded by the amino acid sequence while synthetic polymers in solution were considered as random coils.^5^ By now, many fully intrinsically disordered proteins (IDPs) and intrinsically disordered regions (IDRs) in partially folded proteins were discovered.^6,7^ Their conformational variability is as important for biological function as the well-defined structure of the folded representatives or folded domains.^8−12^ For the other side, advanced concepts in polymer science, such as block copolymers with partial crystallization, go well beyond the concept of random coils.^13−15^ Synthetic polymers are particularly similar to biomacromolecules that do not fold under any conditions, such as polysaccharides^16−19^ and some IDPs.

In general, we have a vast toolbox to characterize the folded segments of macromolecules but fewer tools to discern the diverse conformational states of their unstructured or weakly structured counterparts. In the absence of long-range ordering, scattering methods (SAXS/SANS^20^ and DLS^21^) or labeling-based techniques, such as paramagnetic relaxation enhancement (PRE),^22,23^ cross-linking,^24^ Förster resonance energy transfer (FRET),^25−28^ and pulse EPR dipolar spectroscopy (PDS),^29−32^ are particularly well-suited. Studies on partially structured systems often require an integrative approach, for instance, complementation by NMR and Raman spectroscopy.^33−36^

These systems require an ensemble description of their residual structure. While random-coil synthetic polymers can be characterized by just their radius of gyration, for biomacromolecules, we are often interested in the deviations from random-coil behavior. This applies for instance to polysaccharide dietary fibers (DFs),^37,38^ which are involved in cellular signaling, energy storage, and structural support in cell walls and the glycocalyx. DFs are an important constituent of human diet, as they affect our nutritional biochemistry and hence our health and well-being.^39,40^ From a structural point of view, they represent an intermediate case between genetically encoded heteropolymeric biomacromolecules and random-coil polymers.

PDS can characterize weak order in such macromolecules by providing site-to-site distance distributions^32^ in a range between about 1.5 and 10 nm,^41−43^ usually via nitroxide-based spin labeling.^44−46^ Access to the upper end of the range requires fully deuterated macromolecules and a fully deuterated solvent^47^ because vicinal protons dominate the dephasing of the electron spin and thus limit the maximum dipolar evolution time *t*_max_.^48,49^ If local proton concentration near the labels differs strongly between conformers, apparent distance distributions depend on *t*_max_.^50^ This correlation can provide additional information on the conformational ensemble.^37^

The dependence of signal decay in PDS experiments on local proton concentration near labels can also provide information about intermolecular contacts of the spin-labeled macromolecules or their interaction with small molecules or other types of macromolecules. Such intermolecular interactions affect the conformational distributions and determine the propensity to agglomerate, form gels, or undergo liquid–liquid phase separation (LLPS).^51^ In the specific case of DFs, intermolecular contacts and the agglomeration–dissociation equilibria are strongly affected by the interaction with small molecules.^37,38^ Information on the local proton distributions around spin labels is also of interest for the characterization of soft materials and for the development of dynamic nuclear polarization (DNP) techniques for sensitivity enhancement in NMR.^52^ For DNP polarization agents, one thus obtains information about the nearest protons that are placed just beyond the spectral diffusion barrier. These protons dominate the primary transfer of polarization from the paramagnetic center to the proton spin bath.

The electron signal decay due to the proton spin bath is mediated by electron–proton hyperfine interactions. We have recently developed an approach for elucidating longitudinal spectral diffusion of electron spins in protonated surroundings, based on the relaxation-induced dipolar modulation enhancement (RIDME) technique,^53^ which was originally introduced for measuring electron–electron interactions.^54,55^ We found that the proton concentration around spin labels in homogeneous environments can be reliably determined by global analysis of a series of RIDME measurements with varying mixing times.^53^ Here, we extend this approach to intermolecular hyperfine RIDME (ih-RIDME) for the quantitative characterization of distributions of the local proton concentration in heterogeneous systems. We demonstrate the new approach for spin-labeled polysaccharides. Finally, we propose the combination of hyperfine filtering that underlies ih-RIDME with distance distribution measurements by the double electron–electron resonance (DEER) technique. The proposed experiments provide a means for correlation of local proton concentration around spin-labeled sites to the chain conformation.

We test our approach on a dispersion of barley β-glucan (BBG) soluble dietary fibers in a deuterated D_2_O–D_6_–DMSO solvent mixture.^37,38,56^ By solvent deuteration, we introduce a contrast between the macromolecules that contribute to the ih-RIDME decay and the matrix that does not contribute. This in turn makes the experiment sensitive to the chain conformation of the macromolecules and their interactions with each other. BBG is a mixed-linkage carbohydrate polymer of alternating 1,3- and 1,4-linked β-glucose residues. BBG chains were stochastically spin labeled to achieve about 4.8 spin labels per BBG chain (Figure 1a). They were studied in a free and dye-bound state, with calcofluor white (CalW, Figure 1b) dye being an example of a small molecule that binds to the DFs.

**Figure 1:**
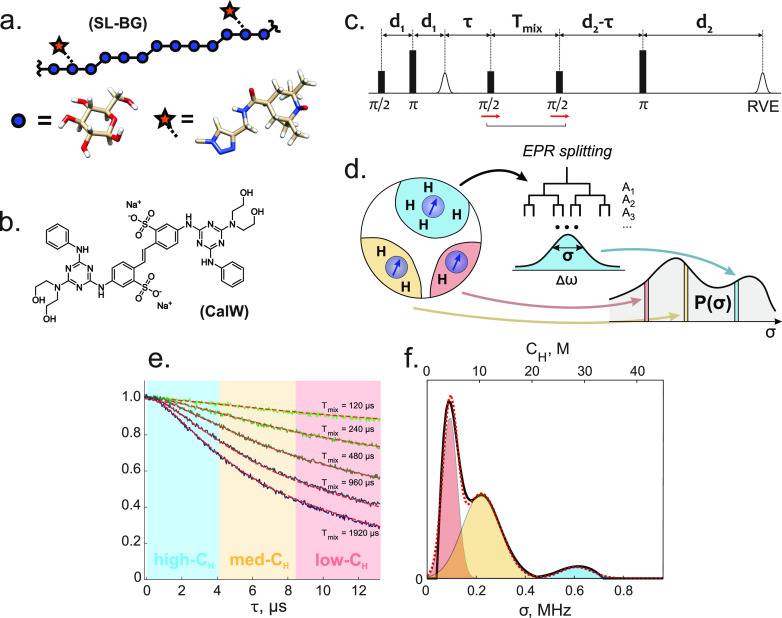
(a) Chemical structure of the spin-labeled BBG chain. (b) Chemical structure of the calcofluor white (CalW) dye molecule. (c) Five-pulse RIDME sequence (RVE stands for “refocused virtual echo”^55^). (d) Schematic representation of the transformation from the EPR resonance frequency, via the distribution of hyperfine shifts, to the distribution of σ values, which are related to the local proton densities (see main text for details). (e) Example of ih-RIDME data set. Five decay traces were recorded at different mixing times (and divided by the trace at *T*_mix_ = 60 μs) for the free BBG sample (black lines). Red lines correspond to the best model-free global fit of the ih-RIDME data. Blue, orange, and red time-range areas indicate different local proton density ranges that have their strongest contribution (more than 14, 7–14, and 4.6–7 M), as described in the main text. (f) Model-free distribution of the local proton densities as fitted in panel e (black) and its decomposition into three Gaussian peaks (dashed red). Individual Gaussian peaks, composing the dashed red curve, are shown as color-filled shapes. The color-code of individual Gaussian peaks indicates the approximate correspondence between the peak positions and the colored time ranges in panel e.

Sensitivity of the RIDME experiment (Figure 1c) to vicinal protons arises from the mixing block consisting of two consecutive π/2-pulses. During this block, electron magnetization is stored along the direction of the external magnetic field *B*_0_. This magnetization takes the form of a polarization grid that is oscillatory on the frequency scale. The finesse of this grid is determined by the defocusing time, τ before the first π/2 pulse of the mixing block. Due to the preceding evolution of electron spin coherence, the electron spins are labeled by the energy of the hyperfine interaction with the closest protons (0.5–3 nm). During the mixing block, the proton bath undergoes continuous quasi-stochastic evolution driven by homonuclear couplings. This causes fluctuation of nuclear spin states. Via the hyperfine coupling, the nuclear spin state is communicated back to the electron spin and, for an ensemble of spins, leads to a stochastic change of the resonance frequency of the electron spin that is called longitudinal spectral diffusion (LSD). This spectral diffusion levels the polarization grid. The echo signal arises from refocusing after the second π/2 pulses have flipped the grid to the transverse plane. Thus, by leveling the grid longitudinal spectral diffusion (LSD) interferes with refocusing and causes a decay of the echo intensity.^53,57^ This signal decay due to LSD adds to the RIDME decay due to electron–electron interactions. The LSD-induced decay can be separated from the influence of electron–electron dipolar interaction by variation of the mixing time *T*_mix_ instead of the dipolar evolution time τ. This way, the LSD phenomenon in the RIDME experiment becomes a sensitive tool to study the proton arrangement around a paramagnetic label or probe.

As illustrated in Figure 1d, an electron spin senses protons up to distances of approximately 3 nm. The electron spin resonance frequency is shifted by a random value, depending on the spin states of all these protons. We approximate the distribution of these shifts by a Gaussian centered at the resonance frequency in the absence of a proton bath. Hence, we characterize the bath by a single parameter—the standard deviation σ of the Gaussian distribution. We call a sample heterogeneous if the proton distribution around the various electron spins in an ensemble differs substantially. Therefore, σ itself is a distributed quantity. In the ih-RIDME experiment, we aim to determine this distribution of σ.

A primary ih-RIDME data set consists of several decay traces measured at different mixing times (*T*_mix_). In this work, we recorded five main traces and an additional sixth reference trace with a short mixing time. The reference trace serves for removal of some artifacts by reference deconvolution, wherein the main traces are divided by the reference trace.^58^ We performed a global fit of the five reference-divided traces, as exemplified in Figure 1e for BBG in the absence of dye molecules or metal ions.

To relate our spin dynamics model to the local proton concentration, we need to introduce two constants. The first constant β relates the τ-dependence of the proton-induced RIDME decay to proton concentration. For a homogeneous proton distribution, we have shown in previous work that this contribution is well-approximated by a Gaussian decay factor *F*(τ) ≈ exp(−*βC*_H_^2^ τ^2^),^53^ Calibration gave β = 7.23 × 10^–5^ (μs mol L^–1^)^−2^. The second constant *D*/σ^3^ characterizes LSD. It is specific for a particular type of protons and allows for normalization of the dipolar frequency correlation function.^53^ Both β and *D*/σ^3^ are invariant with respect to the proton concentration *C*_H_. With these two constants fixed, the set of *T*_mix_-dependent ih-RIDME decays is fully determined by the distribution of the local proton configurations in the vicinity of the paramagnetic probe.

Although proton distribution around an individual paramagnetic center is not necessarily homogeneous, we convert σ (as used in lower horizontal scale in Figure 1f) into an effective local proton concentration (*C*_H_) as an approximation. We do so to invoke chemical intuition (upper horizontal scale in Figure 1f). Our earlier results justify this approximation and showed a simple relation σ ∝ *C*_H_ with the conversion factor 0.0214 MHz L mol^–1^.^53^

By simulating sets of ih-RIDME traces for different proton concentrations in homogeneous samples, we determined time windows where the traces are sensitive to particular concentration ranges (Figure 1e). The steepest ih-RIDME decay is always obtained in the trace recorded with the longest mixing time; here, *T*_mix_ = 1920 μs. The border between the blue region (high *C*_H_) and the yellow region (medium *C*_H_) is set at 4.2 μs. At this time, the ih-RIDME time trace with *T*_mix_ = 1920 μs has decayed 1/*e* times the initial intensity for a proton concentration of 14 M. Accordingly, for *C*_H_ > 14 M, strong effects on signal decay are expected in the time window marked blue in Figure 1e. We designate this as the high-concentration window. Similarly, a time of 8.4 μs (limit between the orange and red windows) corresponds to 1/*e* decay at a proton concentration of 7 M and the end of the red window at 12.8 μs corresponds to *C*_H_ = 4.6 M. These threshold concentrations provide only a rough guidance to the relation between the concentration range and time windows. In general, any local proton concentration provides some contribution to the ih-RIDME decay at all time points.

Remarkably, we found that model-free fits of the distribution of the local proton concentration *p*(*C*_H_) were rather robust with respect to the noise and small experimental imperfections, as illustrated in Figure 1f, although we expected this transformation to be an ill-posed mathematical problem. We tentatively attribute this robustness to the correlated changes in signal decay in the set of five ih-RIDME traces. These changes are governed by a unique parameter *D*/σ^3^ which can be better determined from a multitrace data set as explained in the Supporting Information (SI) (see Figure S7). Because of this, a global fit of the set may be substantially more stable than a discrete inverse Laplace transformation of a single trace. Indeed, when fitting only one or two traces, the *p*(*C*_H_) may be strongly correlated with *D*/σ^3^.

In previous work, we observed partial aggregation of DF chains by size exclusion chromatography (SEC).^38^ DEER measurements and molecular modeling showed that the main source of heterogeneity in such systems is the interchain contacts. This is consistent with our present finding that a model-free fit of *p*(*C*_H_) (Figure 1f) can be closely approximated by a sum of three Gaussian components (Table 1). We can assign the peak with the lowest mean proton concentration to the free BBG chains. We tentatively assigned the two other peaks to two types of aggregates. These types must then have substantially different interchain contact statistics that cause a substantial difference in the mean local proton concentration. As discussed in the SI, we have high confidence in the presence and positions of three components in the fitted model-free *p*(*C*_H_) . In contrast, the widths of these components are uncertain. In particular, the width of the component with the highest local proton concentration is hardly defined (see Table 1 and Figure 2f).

**Table 1:** Numeric Parameters of Individual Gaussian Functions after Approximating the Model-Free Fitted Local Proton Density Distributions from the ih-RIDME Experiments by a Combination of Three Gaussian Peaks: a

System	Mean, M	Width, M	Fraction
Batch 1 (BBG1)	4.35	1.4	0.40
	10.35	3.6	0.54
	28.5	3.0	0.06
BBG1 + CalW (16:1)	4.25	1.4	0.46
	9.80	2.95	0.47
	22.2	6.0	0.07
BBG1 + CalW (1:3)	4.40	1.3	0.47
	10.0	2.75	0.46
	24.2	2.7	0.07
Batch 2 (BBG2)	4.10	1.1	0.34
	10.10	2.25	0.53
	22.2	2.9	0.13
BBG2 + Mg^2+^ (1:20)	4.30	1.25	0.36
	10.20	3.51	0.56
	29.0	4.7	0.08

a Mean, width, and fraction correspond to μ_*i*_, σ_*i*_, and *c*_*i*_, respectively. See graphical representation of these values shown in Figure 2f. For the sample descriptions, the ratio of sugar monomers to small molecules (CalW or Mg^2+^) is indicated in brackets.

**Figure 2:**
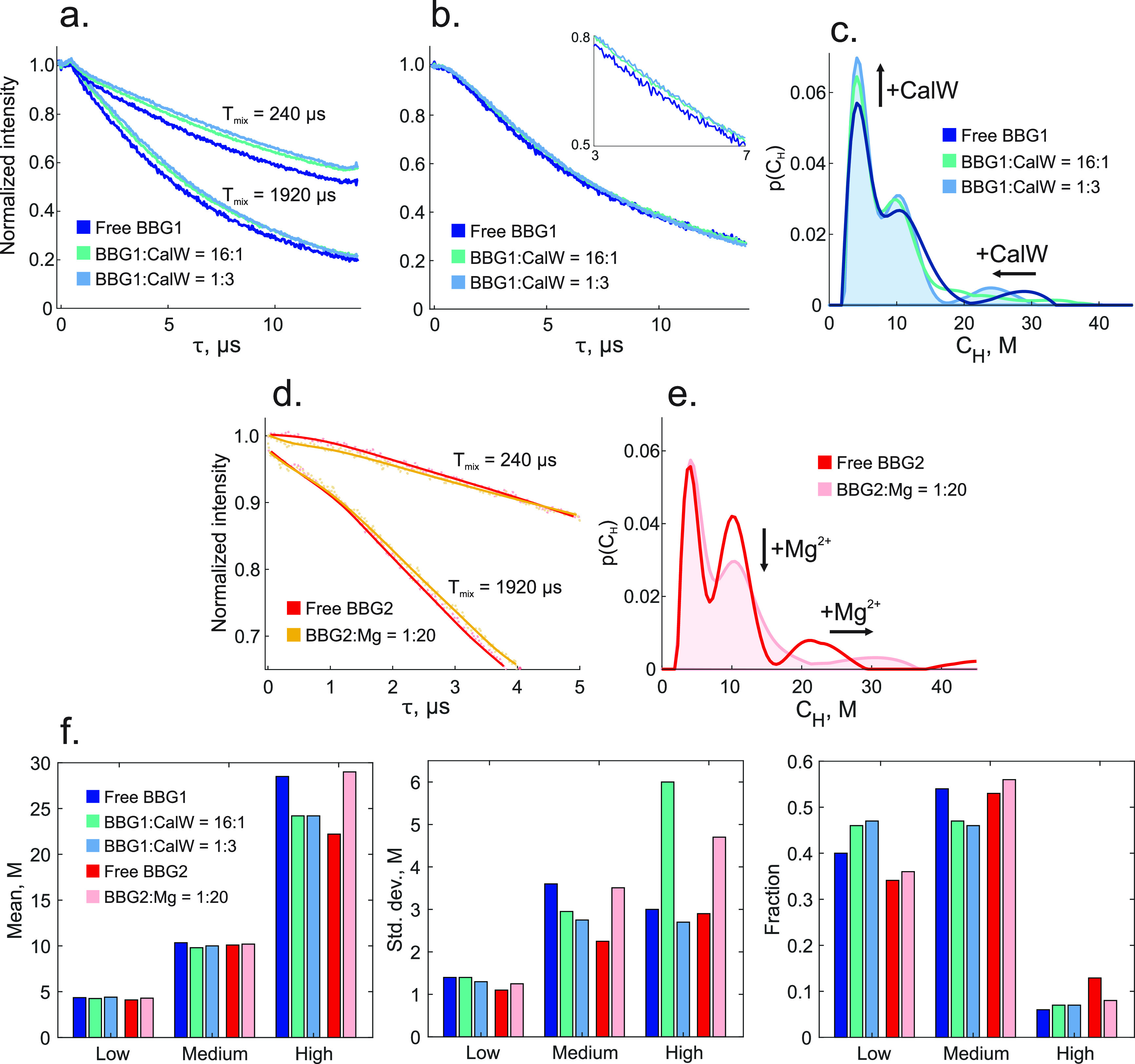
(a) Examples of the recorded ih-RIDME traces for a short (240 μs) and a long (1920 μs) mixing time for free BBG and BBG with two different amounts of CalW added (top three samples in Table 1). (b) Set of ih-RIDME traces from (a) with *T*_mix_ = 1920 μs divided by the reference trace with *T*_mix_ = 60 μs. (c) Comparison of the proton density distributions obtained by the global fit of the set of ih-RIDME traces for the three samples in panel a, i.e. for the top three samples in Table 1. (d) Reference-divided ih-RIDME traces for a short (240 μs) and a long (1920 μs) mixing time of the free BBG and BBG after addition of Mg^2+^(last two samples in Table 1). Traces for *T*_mix_ = 1920 μs are vertically shifted (downward) for better visibility; (e) Corresponding model-free fitted local proton densities for the full data sets for the samples from panel d, i.e., for the last two samples in Table 1. (f) Visualization of the Gaussian parameters after approximating the model-free density distributions in panels c and e with three Gaussian peaks. Numeric values for the Gaussian peak parameters and their statistical weights are given in Table 1.

Next, we checked whether *p*(*C*_H_) changes significantly by the addition of metal ions (Mg^2+^) or dye molecules (calcofluor white, CalW) to the BBG solutions. In the latter case, we expect such changes based on our previous DEER results.^37^ Primary ih-RIDME data (Figure 2a), divided traces (Figure 2b), and fitted proton concentration distributions (Figure 2c) indeed exhibit clear changes upon adding CalW to the BBG solutions (see Table 1 for a sample description and SI for details of the fitting procedure). We attribute the peak at intermediate local proton concentration to weaker chain contacts and the peak at high concentration to aggregates with multiple interchain contacts. With this assignment, it follows that addition of small CalW dye molecules leads to a partial disruption of weak interchain contacts accompanied by an increase in the fraction of free chains. The fraction of strongly aggregated chains fraction decreases as well. However, the aggregates are not completely dissolved. Overall, these data demonstrate that the ih-RIDME approach can track interchain contact statistics with high resolution. Understanding such contact statistics is crucial for characterizing weak interaction of macromolecules, including phenomena like LLPS of proteins.

Upon addition of doubly charged Mg^2+^ ions, which are known to interact with saccharides and other polyalcohols,^59^ we observe changes in ih-RIDME traces as well. As the BBG chains are rather rigid,^60,61^ we expect such ions to enhance the interchain contact probability via cross-linking. Unlike the addition of organic dyes, the addition of Mg^2+^does not introduce new protons. Therefore, an increase of local proton concentrations can arise only from enhanced interchain contacts. Indeed, the experiments reveal a shift of all three contributions to *p*(*C*_H_) toward larger concentrations (Figure 2d,e).

With ih-RIDME experiments, we obtain information on the proton concentration, which is analogous to the information on the electron or nucleon density that can be obtained by small-angle X-ray or neutron scattering experiments (SAXS/SANS). In SANS, deuteration of the solvent or macromolecules is used as well for introducing contrast. There are, however, important differences between the scattering techniques and the label-based ih-RIDME technique. A label-based technique probes *local* structure, in our case in a radius up to about 3 nm from the label. Thus, it does not average over the larger-scale heterogeneity of the sample but rather resolves it. In the case at hand, we studied polydisperse polysaccharides in solutions that contain a substantial fraction of aggregates. In such a situation, scattering curves would be much more difficult to interpret. The ih-RIDME experiment can be performed in a broad range of polymer concentrations, including concentrations where the chains partially overlap. SAXS or SANS data are easy to interpret for interchain distances significantly longer than the average chain length and for dense, near-homogeneous polymer melts. In the intermediate regime, ih-RIDME can provide distributions of the local proton concentration. Another important difference is that ih-RIDME depends on spin labeling. For proteins, where site-directed spin labeling is feasible, the defined and known label sites further enhance resolution and information content. Yet, labeling raises the concern of distortion of the native ensemble. Further, ih-RIDME data are acquired in a glassy frozen solution at cryogenic temperatures, whereas SAXS and SANS or FRET can be applied at ambient temperature. Therefore, the various techniques complement each other. Integration of small-angle scattering data, FRET data, ih-RIDME data, and DEER data in ensemble modeling should enable more detailed characterization of complex systems than reliance on only a single technique.

The BBG samples that we studied here feature heterogeneity due to polydispersity of the carbohydrate and aggregation. These phenomena cause variation of the local concentration of sugar protons that we can characterize by *p*(*C*_H_) . To verify our results, we need some independent information on *p*(*C*_H_) . In the absence of a complete multiscale model, which is currently out of reach, we cannot obtain this information from SAXS or SANS experiments. Instead, we compared our ih-RIDME data to Monte Carlo (MC) simulations of single BBG chains. This way, we can at least check the assignment of the resolved peak at low *C*_H_ to isolated chains as well as its position. The MC ensemble we used was previously verified to reproduce the correct DEER signal recorded on the BBG samples at very low concentration.^38^

The distributions *p*(*C*_H_) predicted from the MC-simulated ensemble of spin-labeled BBG chains are in rather good agreement with the ih-RIDME result. In particular, the mean value of the lowest-concentration Gaussian peak in the ih-RIDME distribution and the mean value of the local proton concentrations in the MC-modeled ensemble are close to each other (Figure 3a; see SI for calculation details). Examples of BBG conformations with the lowest and highest local proton concentrations are shown in Figure 3b. Note that the MC-modeled distribution is slightly skewed toward higher local proton concentrations, showing that our model of three Gaussian components is somewhat deficient. Nevertheless, the MC results predict much weaker contributions at proton concentrations above 8 M than we see in *p*(*C*_H_) derived from ih-RIDME traces. This finding supports our assignment of the peaks at higher concentration to aggregates. In the future, ih-RIDME could aid further development of molecular dynamics (MD) modeling, which often requires adjustment of force-field parameters for disordered systems. Experimental distributions of local proton concentration may provide valuable information for benchmarking such parameter sets.

**Figure 3:**
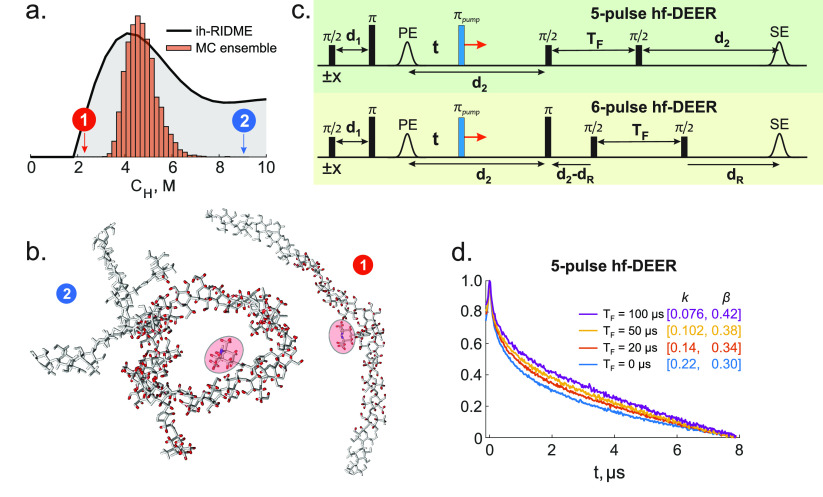
(a) Low proton density part of the local proton density distribution fitted from ih-RIDME data for the free BBG (top sample in Table 1), black curve, and MC simulation of proton densities for a conformational ensemble of isolated BBG chains (histogram). The arrows show examples of spin labels in different local chain structures: (1) example of the most extended conformation with the lowest local *C*_H_, and (2) example of the most compact conformation encountered in the MC ensemble, with the highest local *C*_H_ near the intrachain contact. (b) Atomic representation of the BBG conformations (1 and 2) indicated in panel a. The position of the spin label is shown in pink. The protons in a spherical shell of 2.5 nm radius near the spin label are highlighted in red, and atoms outside the shell are in gray color. The magnified versions of the structures can be found in SI Figure S10. (c) Upper panel: pulse sequence of the 5-pulse hyperfine-filtered DEER (hf-DEER) experiment as a modification of standard 4-pulse DEER where the last π-pulse is replaced by two π/2-pulses. Lower panel: pulse sequence of the 6-pulse hf-DEER experiment which originates from the standard 4-pulse DEER with a hyperfine filtering block inserted within the last transverse evolution period. (d) Recorded traces of 4-pulse DEER (*T*_F_ = 0 μs) and 5-pulse hf-DEER for the free BBG sample (top sample in Table 1). The traces are rescaled to the same vertical split between the signal maximum and minimum points to visualize the hf-DEER trace shape evolution with a changing mixing time. Numbers in brackets display optimal parameters of stretched exponential fitting of traces in format [*k*, β] → exp(−*k*·(*t*/μs)^β^).

As mentioned above, ensemble modeling of heterogeneous macromolecular systems requires integration of different types of information. In particular, it is of interest to integrate information on local proton concentration with information from longer-range label-to-label distance distributions that can be obtained by DEER. This endeavor would profit from an experiment that correlates the two distributions, as such correlation would shed light on contact statistics for differently extended chains. We have previously established an approach to observe the heterogeneity in such systems by applying a *T*_m_ filter in DEER measurements.^37,38^ This previous approach provides only qualitative information, whereas the ih-RIDME experiment can provide quantitative information about the distribution of local proton concentrations. Figure 3c presents two pulse sequences, where a RIDME block is inserted as a filter into the DEER pulse sequence. In such hyperfine-filtered DEER (hf-DEER) experiments, the electron–electron dipolar evolution is stopped during the RIDME block. The experiment is performed at temperatures where electron spin flips during the mixing block are negligible, so that no RIDME effect in the narrower sense contributes to the data. However, the spectral diffusion processes are active. Therefore, signals from labels with a larger local proton concentration are attenuated more strongly with increasing filtering time *T*_F_ than those from labels with smaller local proton concentration. The accessible range of filtering times is limited by the longitudinal electron relaxation *T*_1_. Since typically *T*_1_ ≫ *T*_m_, hf-filtered DEER provides better resolution than *T*_m_-filtered DEER. The 6-pulse hf-DEER experiment shown in the lower panel of Figure 3c offers flexibility for setting the finesse of the magnetization grid that determines sensitivity of the RIDME block to spectral diffusion. This flexibility is introduced by refocusing delay *d*_R_. The shorter the *d*_R_, the larger the period of the grid on a frequency scale. In the case of the five-pulse hf-DEER experiment in the upper panel of Figure 3c, the RIDME block starts at maximum spin ensemble dephasing, corresponding to the finest magnetization grid (*d*_R_ = *d*_2_) that is feasible at the given interpulse delay *d*_2_. Thus, for a sequence of given maximum duration, 5-pulse hf-DEER has a maximum sensitivity toward LSD which may also lead to the highest signal losses. Figure 3d shows a series of 5-pulse hf-DEER traces. The slower decay of the DEER traces at longer *T*_F_ implies a positive correlation between local proton concentration and local electron spins density. For a shorter trace length of 4.2 μs instead of 8.2 μs, corresponding to less finesse of the grid, the effect of hyperfine filtration is weaker as discussed in the SI.

In order to quantify the correlation, we needed to establish a global analysis of the set of decay traces in terms of a bivariate distribution. We conjecture that a numerically robust solution to this problem requires independent determination of the univariate marginal distributions of electron–electron distances and local proton concentrations. The BBG samples studied here are not suitable for this because they are stochastically spin-labeled. They therefore give rise to multielectron–spin distributions of dipole–dipole couplings, which we cannot unambiguously convert to pair distance distributions. Thus, we defer the development of the correlation experiment to future work with more suitable model samples such as site-directed spin-labeled proteins or nucleic acids.

To conclude, we described an approach for quantifying the heterogeneity of local proton concentration around spin-labeled sites in soft matter or solutions of macromolecules by using the pulse EPR technique ih-RIDME. Transformation of the ih-RIDME data to distributions of local proton concentration is numerically stable when based on a global fit of a series of time traces. Further, the ih-RIDME block is combinable with DEER measurements of electron–electron distance distributions. Future work in this direction may provide an approach to the correlation of electron–electron distances to local proton concentrations in terms of a bivariate distribution. At the current stage, we found ih-RIDME to be applicable to soluble carbohydrates. By analogy, we expect it to be applicable to studies of biomolecular condensates formed by LLPS. We hope that our methodology will also aid the development of DNP methods, where the local proton distribution near a paramagnetic polarizing agent determines the efficiency of polarization transfer to bulk protons. In general, we expect ih-RIDME to complement scattering and FRET techniques in studies of systems that are neither completely disordered nor completely ordered.

## Supporting Information Available

Synthetic, experimental, and numerical procedures and characterization data for all new compounds (PDF)
Transparent Peer Review report available (PDF)
