# Supplementary material for: Quantification of Distributions of Local Proton Concentrations in Heterogeneous Soft Matter and Non-Anfinsen Biomacromolecules
Source: J Phys Chem Lett. 2024 May 17;15(21):5625–32. doi: 10.1021/acs.jpclett.4c00825 (PMC11145652; doi:10.1021/acs.jpclett.4c00825)
Supplement: Supplementary file 1 — jz4c00825_si_001.pdf [file jz4c00825_si_001.pdf]

Electronic Supporting Information:

Quantification of Distributions of Local Proton Concentrations in Heterogeneous Soft Matter and non-Anfinsen Biomacromolecules

Sergei Kuzin,[*,†] Dario Stolba,[†] Xiaowen Wu,[‡,¶] Victoria N. Syryamina,[‡,§] Samy Boulos,[‡] Gunnar Jeschke,[†] Laura Nyström,[‡] and Maxim Yulikov[*,†]

†*Department of Chemistry and Applied Biosciences, ETH Zurich, Vladimir-Prelog-Weg 2, 8093, Switzerland*

‡*Department of Health Sciences and Technology, ETH Zurich, Schmelzbergstrasse 9, 8092, Switzerland*

¶*Max Planck Institute of Colloids and Interfaces, Potsdam, Germany, 14476*

§*Voevodsky Institute of Chemical Kinetics and Combustion, Novosibirsk, Russia, 630090*

E-mail: sergei.kuzin@phys.chem.ethz.ch; maxim.yulikov@phys.chem.ethz.ch

Contents

Sample preparation and characterization S2

EPR measurements S7

Numerical fitting of ih-RIDME data S10

Molecular modeling S14

References S19

Sample preparation and characterization

Materials

Chemicals: 4-carboxy-2,2,6,6-tetramethyl-piperidin-1-oxyl (4-carboxyl-TEMPO, 97 %), 2-propynylamine (98 %), N,N-dimethylpyridin-4-amine (DMAP, 98 %), N-(3-dimethylaminopropyl)-N′-ethyl-carbodiimid-hydrochloride (EDC.HCl, commercial grade), triethylamine (TEA, 99 %), anhydrous lithium chloride (LiCl, 99 %), Copper(I) bromide (CuBr, 98 %), N, N, N′, N′, N″, N″-pentamethyldiethylenetriamine (PEDMTA, 99 %), p-toluenesulfonyl chloride (TsCl, >99 %), and potassium bromide (KBr, FT-IR grade) were purchased from Sigma-Aldrich (St. Louis, MI, USA). The barley β-glucan (BBG) (low viscosity, Lot 100401, M_w 179 kDa, $\approx$ 95 % purity) was purchased from Megazyme. Solvent used in synthetic works: Dichloromethane (DCM, 99.8 % Extra Dry over molecular sieves), dimethylacetamide (DMA, 99.5 % Extra Dry over molecular sieves), and Dimethyl sulfoxide (DMSO, 99.7 % Extra Dry over molecular sieves) were purchased from Acros (Geel-Belgium) and used without further purification. Water used in this work was purified using a Millipore MilliQ system (Billerica, MA, USA). Deuterium oxide (D_2O, 99.9 %) and dimethylsulfoxide-d6 (DMSO-d6, 99.9 %) used in NMR and pulse EPR were purchased from Sigma-Aldrich (St. Louis, MI, USA). Dialysis membranes used for sample purification made from regenerated cellulose with MWCO 12-14 kDa (25 Å; 29 mm) were supplied by SERVA (Heidelberg, Germany).

Synthesis of spin-labeled BBG

The preparation of spin-labeled BG (SL-BG) was followed based on our previous work.[1]

Synthesis of the alkynyl-TEMPO (compound 1)

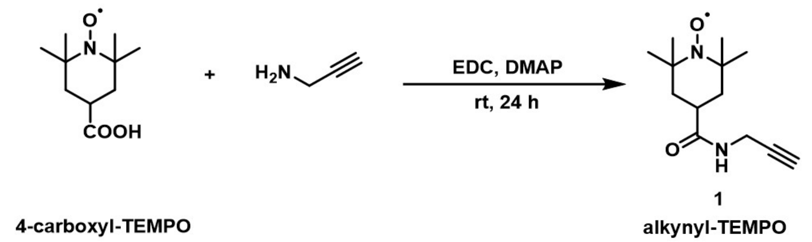

Figure S1: Synthesis of alkynyl-TEMPO.

4-Carboxyl-TEMPO (200 mg) was dissolved in 10 mL of anhydrous DCM, 1 equiv. of 2-propynylamine was added followed by 0.1 equiv. of DMAP, and 1 equiv. of EDC (Scheme 1). The resulting mixture was stirred at room temperature for 24 h. The reaction was quenched by ice water, and the organic phase was washed with saturated NaCl solution and water three times each. DCM was removed in a rotary evaporator. Finally, the resulting crude oil underwent silica gel flash column chromatography eluted with DCM:MeOH = 50:1 to get 180 mg of compound 1 as a light-yellow powder (yield 75 %). The product was characterized by HRMS (High-Resolution Mass Spectrometry). MS (m/z) (ESI, MeOH) calculated for $[C_{13}H_{21}N_2O_2]^+$ is 237.1603; found 237.1606.

Synthesis of Tosyl-substituted BG (BG-OTs)

Barley β-glucan was dried under vacuum at 100°C in an oil bath overnight before use. Then, 250 mg dried BG was dissolved in 30 mL 3 % anhydrous LiCl/DMA solution at 100°C under vigorous stirring until fully dissolved. After cooling down, the resulting solution was placed in an ice bath with stirring and 2 mg of TEA in 1 mL of DMA was added. 42 mg (0.18 equiv. to monosaccharide unit) of TsCl dissolved in 2 mL of DMA was added dropwise into the above solution followed by stirring in ice bath for 30 min and then under room

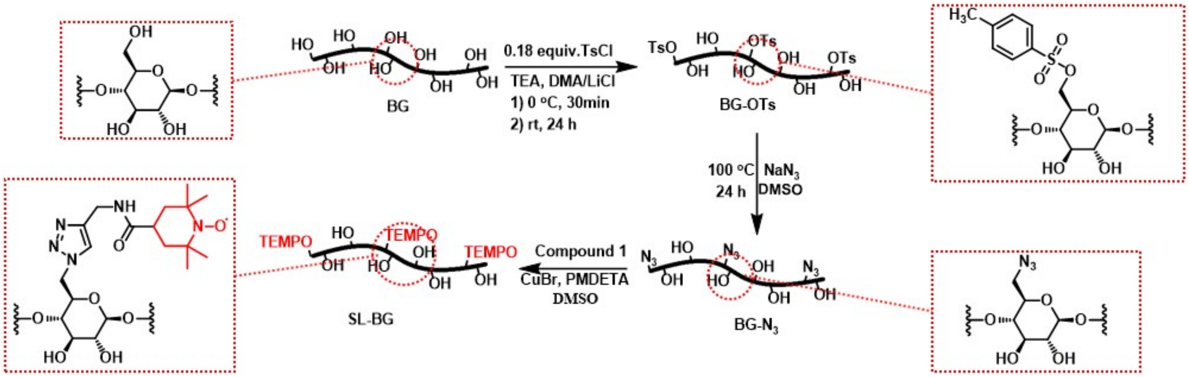

Figure S2: Illustration of the synthetic pathway to produce SL-BG. The inserts show the structures of the chemical modifications on the monosaccharide repeating unit (only the predominant products with substitution at C6 are shown).

temperature for 24 h. The resulting solution was diluted with 300 mL of water and purified by dialysis against water (3 times 5 L) for 3 days. A white cotton-like product yielded after lyophilization (recovery yield 96 %). The final product was confirmed by ^{1}H NMR with low field (7.76 ppm and 7.78 ppm) of ^{1}H signal from the aromatic ring as well as the high field (2.41 ppm) signal from -CH_3 from tosyl (Figure S3, red), and by FT-IR with an extra absorption of the aromatic ring at 1750 cm^{-1} compared with the native BBG (Figure S4A).

Synthesis of N_3-substituted BG (BG-N_3)

100 mg of BG-OTs were dissolved in 10 mL of anhydrous DMSO, followed by the addition of 20 mg of NaN_3. The solutions were stirred at 100 °C for 24 h. Afterwards, the solution was diluted with 100 mL of water and purified by dialysis against water (3 times 5 L) for 3 days. A white cotton-like solid product was yielded after lyophilization (recovery yield: 80 %). The products were confirmed by the disappearing signal of aromatic and methyl protons from tosyl in ^{1}H NMR spectrum (Figure S3, blue) as well as FT-IR spectrum (Figure S4A).

Synthesis of spin labelled BG (SL-BG)

50 mg of N_3-substituted DF was dissolved in 5 mL dried DMSO in nitrogen atmosphere. 10 mg alkynyl-TEMPO (1), 2 mg CuBr and 0.1 mL N, N, N′, N′, N″, N″-pentamethyldiethylenetriamine (PMDETA) were added under stirring, respectively. The solutions were stirred for 3 days at room temperature in the dark. The resulting product solution was quenched and diluted with 10 times cold water and dialysis against water (3 times 5 L). Then the solution was washed with DCM 3 times. Finally, a solid product was yielded after lyophilization (recovery yield: 93 %).

Samples preparation for pulse EPR

For *batch 1* sample, SL-BG was suspended in D_2O at a concentration of 5 $mg\,mL^{-1}$ and heated up at 80 °C under vigorous stirring for 4 h. The SL-BG D_2O solution was used as the stock solution for batch 1. For *batch 2* sample, the same concentration of SL-BG suspension as batch 1 was prepared, but heated up at 40 °C until dissolved (approximately 2 h). This SL-BG D_2O solution was used as the stock solution for batch 2. The ligands (CalW or Mg^{2+} in the form of $MgCl_2$) dissolved in either DMSO-d6 or D_2O were added into above mentioned stock solution and mixed using a vortexer. The final concentration of SL-BG was kept constant at 1030 $\mu mol\,L^{-1}$ of monosaccharide (0.19 $mg\,mL^{-1}$; bulk spin concentration 32.5 $\mu mol\,L^{-1}$). The ratio mentioned in the main text refers to the ratio between monosaccharide residues and ligands. For example, BBG+CalW(16:1) means monosaccharide concentration is 1030 $\mu mol\,L^{-1}$ and CalW concentration is 64.4 $\mu mol\,L^{-1}$. For all low-temperature pulse measurements, 35 µL of the mixture solution were transferred into 3 mm quartz tubes (outer diameter 2.95-3.05 mm). The samples were frozen rapidly by immersing the sample tubes into liquid nitrogen to ensure a glassy frozen matrix formation. Between EPR measurements, samples were stored at –80 °C.

Proton nuclear magnetic resonance (^{1}H-NMR) spectroscopy

The intermediate product BG-OTs and BG-N_3 were characterized by NMR spectroscopy (Bruker AVANCE III-400 spectrometer 400 MHz, Ettlingen, Germany) and compared with native BG. 10 mg samples were dissolved in 700 µL DMSO-d6 and transferred into NMR tubes. NMR spectra were acquired at room temperature at 400 MHz. Data processing was carried out on MestReNova 14 (Mestrelab Research SL, Santiago de Compostela, Spain).

Fourier Transform Infrared (FT-IR) Spectroscopy

Samples were mixed with pre-dried potassium bromide (KBr) at a concentration of 1 % (w/w), milled in a fine powder until no crystallites were discernible anymore and pressed to a transparent tablet. A control sample using pure KBr was used as the background that was subtracted from each sample spectrum. Samples were analysed by Varian 640 FTIR spectroscopy (Agilent Technologies, Santa Clara, California). The IR transmittance was scanned over the range from 4000 to 400 cm^{-1} with a resolution of 2 cm^{-1} at room temperature and averaged over 64 scans.

Room temperature CW EPR measurement

The SL-BG and standard free TEMPO in H_2O solutions were measured with continuous wave (CW) EPR by a benchtop ESR Spectrometer MiniScope MS300 (Magnettech, Berlin, Germany) equipped with a frequency counter FC300. Setup for measurements: the microwave frequency was 9.4 GHz, B_0 was 335 mT, sweep width was 10 mT, number of measured field points was 4096, sweep time was 30 s, magnetic field modulation was 0.1 mT, microwave attenuation was 22 dB, and number of scans was 3. TEMPO in H_2O (2 $\mu mol\,L^{-1}$) was used as a reference standard for the EPR instrument.

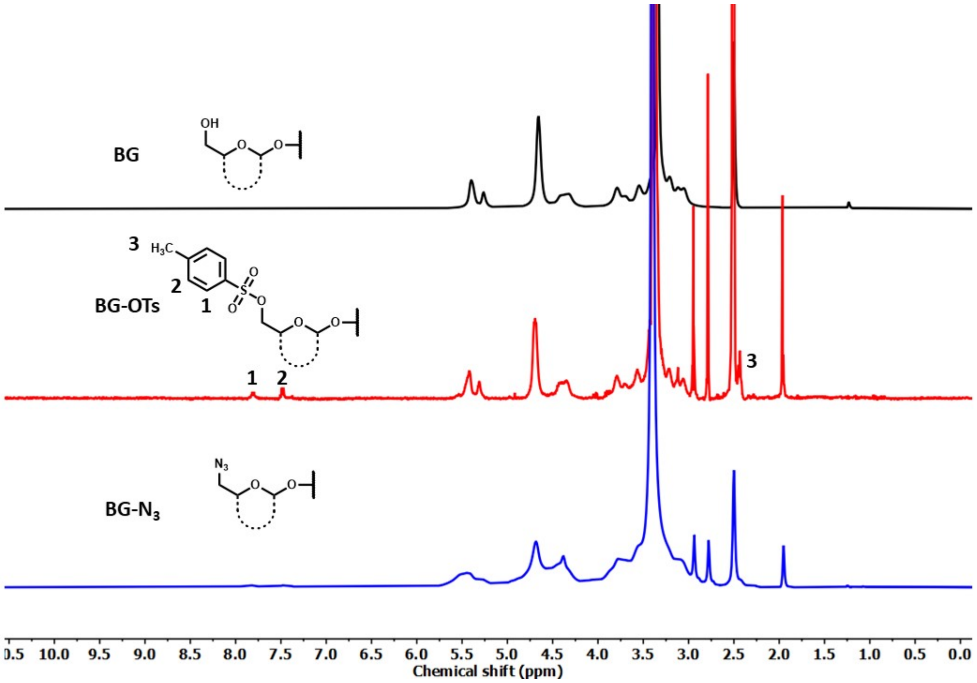

Figure S3: ^{1}H NMR spectra (400 MHz, DMSO-d6) of native BG, BG-OTs, and BG-N_3. The inserted numbers are the assigned peaks of the tosyl group in BG-OTs.

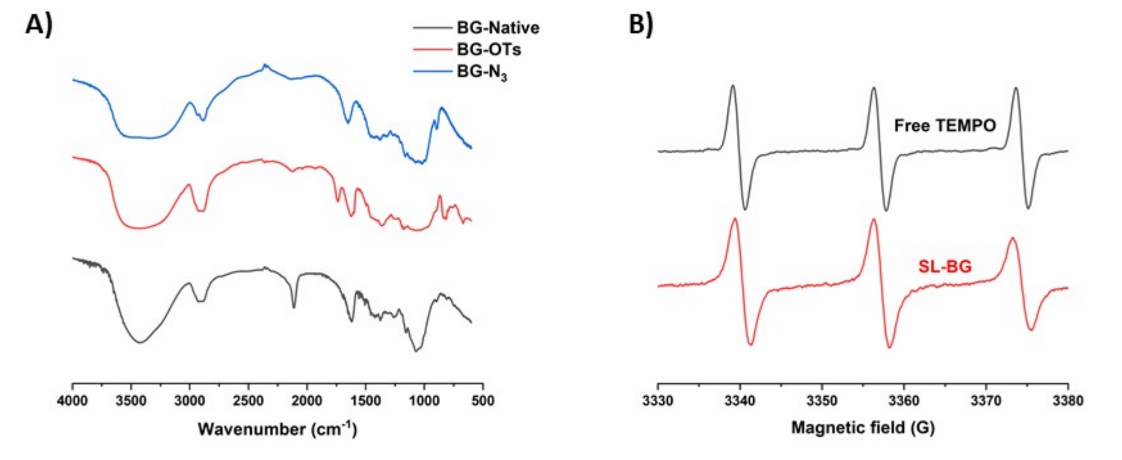

Figure S4: A) FT-IR spectra of native BG, BG-OTs and BG-N_3; B) Room temperature CW EPR spectra of free radical TEMPO (black) and SL-BG (red) measured in water.

Pulsed EPR measurements

All pulsed EPR measurements were conducted at a home-built spectrometer at Q band ($\nu_{MW} = 34.5$ GHz) in a home-built resonator for oversized tubes (outer diameter 3 mm).[2] If

not stated otherwise, the temperature was set to 25 K. This temperature was selected after optimization in order to suppress the T_1-relaxation of the nitroxide spin labels located on the same chain or in the same interchain complex during the mixing block in RIDME. At higher temperatures, the shape of RIDME traces may be sensitive to temperature fluctuations.

Echo-detected EPR spectra are recorded as the intensity of primary echo ($\pi/2 - t - \pi - t -$ echo) versus the magnetic field. Delay t was set to 0.4 µs. All relaxation and ih-RIDME measurements were conducted at the maximum point of the EPR spectrum (Figure S5a).

RIDME experiments were performed with the following parameters: $t_{\pi/2} = 12$ ns, $t_\pi = 24$ ns, $d_1 = 0.4$ µs, $d_2 = 14.2$ µs, $T_{\text{mix}} = 60, 120, 240, 480, 960, 1920$ µs. The meaning of the delays d_1 and d_2 in terms of pulse sequence can be found in the main text (Figure 1c). The mixing block was shifted with a step of 40 ns. Additionally, a protocol for deuterium ESEEM averaging was used[3] with 8 steps of 16 ns of increment. The shortest mixing time (also referred to as reference mixing time, $T_{\text{mix}}^{\text{ref}}$) is chosen to guarantee 95% of transverse relaxation during the mixing block, as shown in Figure S5b. The rest of the values form a geometric series with a common ratio of 2. The delay d_2 influences the minimal proton concentration to be properly detected. This delay must be long enough so that the ih-RIDME trace of spin labels with the local proton concentration of interest achieves $1/e$ decay.

Hyperfine-filtered double electron-electron resonance (hf-DEER) traces were recorded for BBG1 (see Table 1) at 50 K. The duration of all pulses was set to 12 ns. The pump pulse's (blue bar in Figure 2b) frequency corresponds to the maximum of the EPR spectrum. The frequency of the observer pulses (black bars) is shifted by -100 MHz relative to that of the pump pulse. The position of the filtering block in the sequence allows for additional tuning of the filtration effect (FE). The closer it is to the detection event, the smaller the filtration depth for the spin labels in low-protonated environments. In the presented results the filtering block replaced the last refocusing π-pulse (referred here to as the 5-pulse version) ensuring the largest FE for all spin packets. We have tested $d_2 = 4.2$ and 8.2 µs. The duration of the filtering block (T_F) was scanned in the range 0 to 100 µs featuring the

increasing effect at longer waiting times. $T_F = 0$ is understood as the absence of the filtering block, therefore, the pulse sequence corresponds to 4-pulse DEER. Data was fitted with a stretched exponential function $V(t) = \exp(-k(t/\mu s)^{\beta})$. The value of k decreases due to the filtration of the electron-electron dipolar interactions. Parameter β increases due to the inhomogeneity of the filtration effect. This is a sign of a specific correlation between short electron-electron distances and dense protonic parts in the structure of a single polymer chain or an inter-chain complex. If d_2 is chosen too short, one may not achieve a necessary phase difference between various labels (Figure S6). Therefore, a homogeneous filtration only takes place leading to a decrease of modulation depth, while changes in the shape of the decay trace are hardly detectable.

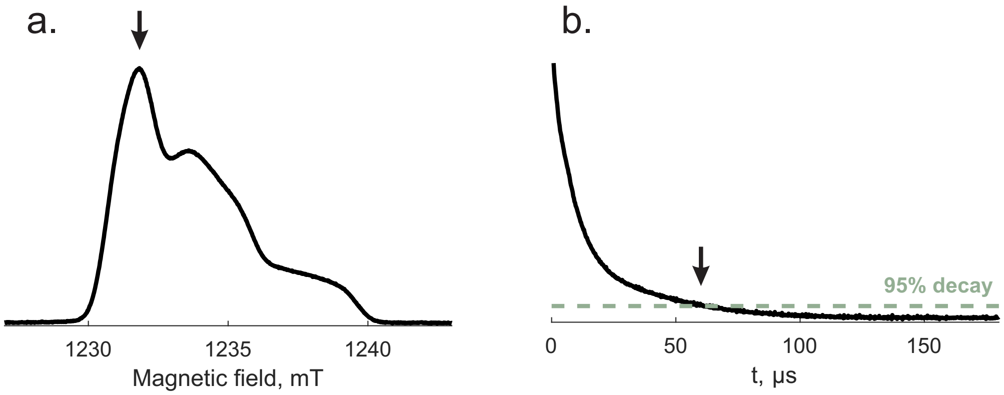

Figure S5: a. Echo-detected EPR spectrum of the batch 1 free BBG sample (top sample in the Table 1, main text). An arrow indicates the field position for the relaxation and the ih-RIDME measurements. b. Transverse relaxation trace of the same free BBG sample. An arrow emphasizes the point of 95% decay which corresponds $t \approx 60$ μs. This time was used as the mixing time in the reference ih-RIDME traces.

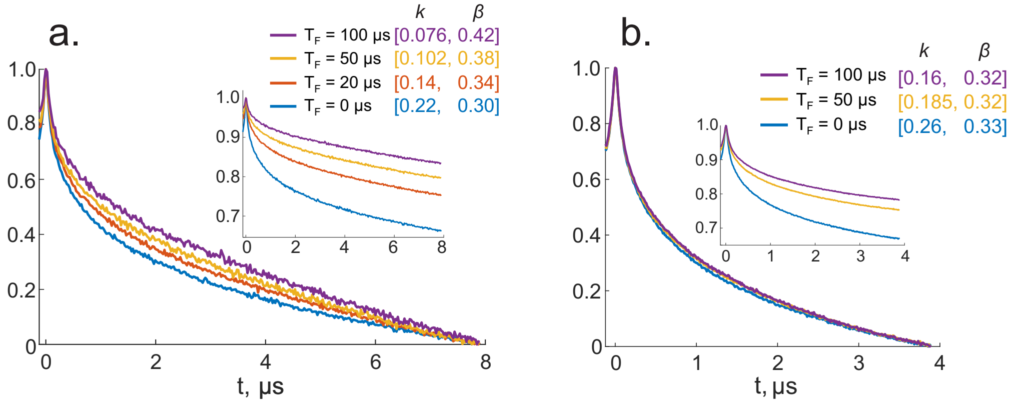

Figure S6: Normalized hf-DEER traces with (a.) $d_2 = 8.2$ µs (b.) $d_2 = 4.2$ µs. Normalization is done by linear scaling to match the maximum and the last points of all traces. The inset shows the raw data. Numbers in brackets display optimal parameters of stretched exponential fitting of traces in format $[k, \beta] \rightarrow \exp(-k \cdot (t/\mu s)^{\beta})$.

Numerical fitting of ih-RIDME data

ih-RIDME fitting model

The model of the decoherence in ih-RIDME trace is based on the description of quasi-stochastic fluctuation of a spin state of the proton reservoir around the spin label. Even if the molecular motion is frozen, the nuclear magnetic degree of freedom remains unconstrained leading to a distribution of possible local hyperfine fields (ω) generated by protons in the vicinity of the electron spin. This distribution is referred to as a (local) hyperfine spectrum $\rho_{\text{loc}}(\omega)$. Due to a large number of nuclear states (2^N with N being the number of close protons), the hyperfine spectrum may be assumed quasi-continuous and approximated by a Gaussian function $\rho_{\text{loc}}(\omega) = \exp(-\omega^2/2\sigma^2)/(\sqrt{2\pi}\sigma)$. Therefore, the local proton density is encoded by a single value σ that has a frequency unit. This value is not unique but is determined by a spatial arrangement of protons. In particular, we found $\sigma \propto C_H$ for homogeneous solutions where the proton concentration is well-defined.[4] In an ensemble of stochastically labeled polymer chains, proton distributions in space may vary a lot for different labels. We take this into account by introducing a non-negative density distribution function $p(\sigma)$. Hence, we assume for the ih-RIDME data the following structure

$$V(\tau; T_{\text{mix}}) = \int_{\sigma_{\min}}^{\sigma_{\max}} p(\sigma) V_{\sigma}(\tau; T_{\text{mix}}) \mathrm{d}\sigma \tag{S1}$$

or in terms of local proton concentrations

$$V(\tau; T_{\text{mix}}) = \int_{C_{\min}}^{C_{\max}} p(C_H) V_{C_H}(\tau; T_{\text{mix}}) \mathrm{d}C_H \tag{S2}$$

where V_σ and V_{C_H} mean the ih-RIDME traces corresponding to a given σ, respectively, C_H. The distributions $p(C_H)$ and $p(\sigma)$ are normalized to unity.

The integration limits have natural boundaries. Namely, $C_{\min}$ must be non-negative, whereas a value of zero corresponds to detached spin labels in the deuterated solvent. The $C_{\max}$, in turn, cannot exceed the proton concentration in crystalline glucose. Considering that the protons of the hydroxyl groups are exchanged by deuterons, the limiting value is 62 $\mathrm{mol\,L^{-1}}$.

Previously,[4] the ih-RIDME traces were presented as products $V_{C_H}(\tau; T_{\text{mix}}) \approx F_{C_H}(\tau) R_{C_H}(\tau; T_{\text{mix}})$. The first factor was approximated with a Gaussian decay $F(\tau) \approx \exp(-\beta C_H^2 \tau^2)$ where parameter β was set to $7.23 \cdot 10^{-5}$ $\mathrm{L^2/mol^2/\mu s^2}$ for all systems. D/σ^3, which parameterizes the shape of the second contribution, was optimized in this work to 1.35 $\mathrm{ms^{-1}}$.

Details on numeric procedure

For the numeric fitting, a home-written MATLAB (*www.mathworks.com*) script was used. The equation (S2) was discretized

$$V(\tau; T_{\text{mix}}) = \sum_{j=1}^{N} p(C_j) V_{C_j}(\tau; T_{\text{mix}})\,. \tag{S3}$$

The concentration axis was also uniformly discretized with the following parameters: $N = 100, \min(C) = 0\ \mathrm{mol\,L^{-1}}, \max(C) = 45\ \mathrm{mol\,L^{-1}}$. The density distribution function was normalized to 1 $\sum_{j=1}^{N} p(C_j) = 1$. The τ-axis and the T_{mix} were the same as in the experimental

data.

The procedure was then based on the non-linear gradient least-squares global fitting of the set of experimental reference-divided traces ($V(\tau; T_{\text{mix}})/V(\tau; T_{\text{mix}}^{\text{ref}})$). The elements of the distribution array were varied. At each optimization step, the normalization and the non-negativity conditions were ensured. No regularization technique was used for $p(C_H)$. Each dataset was fitted with 5000 steps of gradient optimization.

Importance of global fitting

In this section, we discuss the stability of the ih-RIDME data fitting. This is achieved, at the core, due to a global analysis of T_{mix}-series. Indeed, changes in a single trace are usually not enough to obtain a robust fit. If only one trace is considered, its variations may be often satisfactorily explained by changes of either $p(C_H)$ or D/σ^3. Processing of multiple traces disentangles these two parameters.

Prior to the consistent investigation of the proton density distributions, we performed optimization of (D/σ^3) via a relaxed scan. This step can be usually also done with a smaller number of optimization steps. The rmsd slice is in Figure S7a and the optimal value is 1.35 ms^{-1}. The shape of the rmsd-curve around the minimum is pronounced excluding the correlation with the proton density distribution. Although the latter has more degrees of freedom, especially if a smoothness regularization is not applied, the time-domain fit is never satisfactory when D/σ^3 is chosen wrong. We demonstrate this by repeating the fit using deviating values (Figure S7b and c). At lower values, the fit outputs an "extended" fork and misdetermines the low-concentration peak. Oppositely, with the overestimated D/σ^3, the "shrunk" fork is found in the time domain and the high-concentration peak is distorted. Note that even in such extreme cases the trimodal structure of the distribution is recognizable. Overall, we find it promising that the shape of the distribution cannot compensate for a poor choice of model parameters. This indicates robustness of the fitting result.

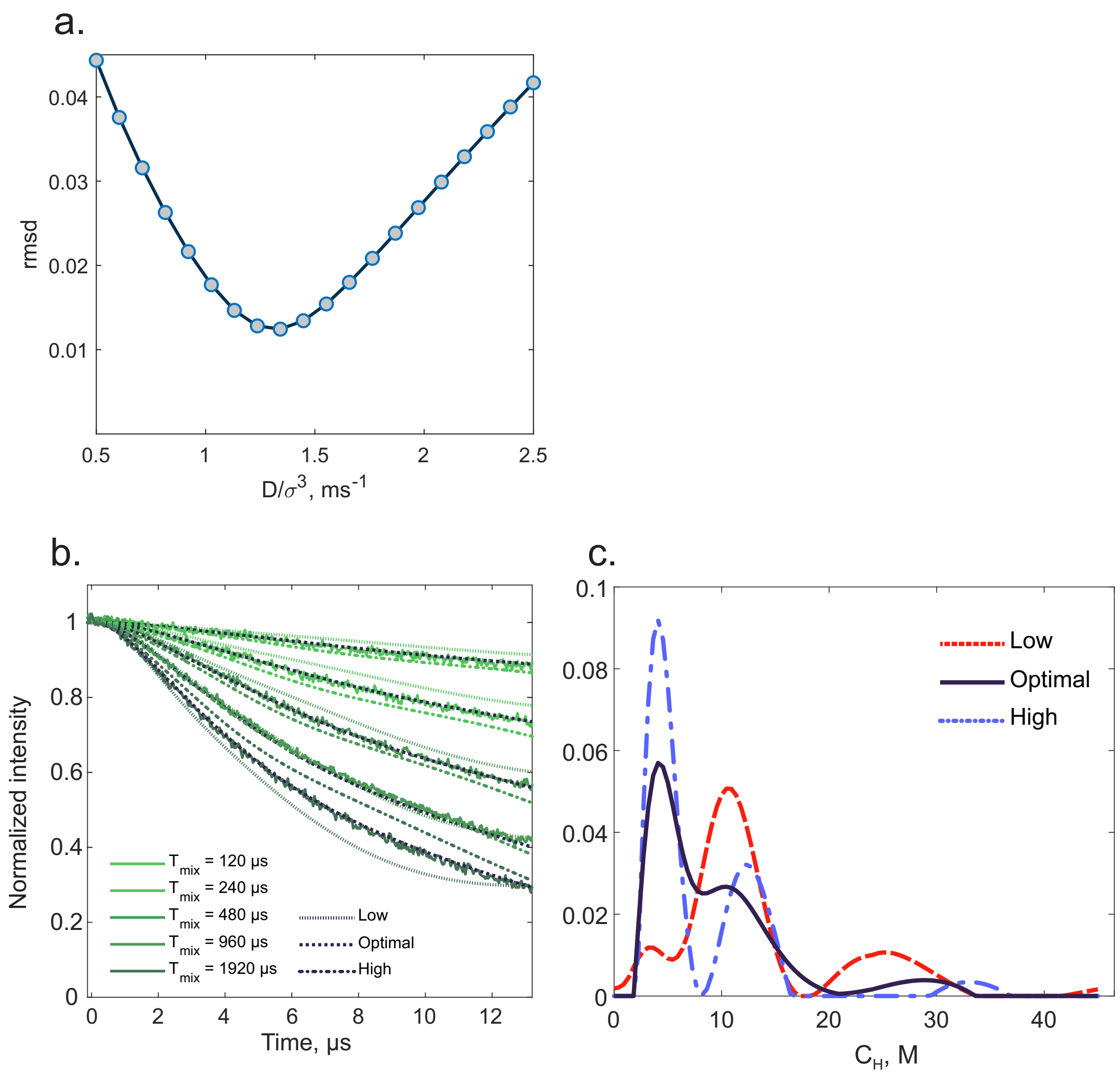

Figure S7: (a) Relaxed scan of (D/σ^3); (b) Fitting results with a fixed (D/σ^3) of low, optimal and high value (respectively, 0.5, 1.35 and 2.5 ms^{-1}); (c) Corresponding fitted local proton concentration distributions.

Convergence of distribution moments

For the data fitting, we used a home-written MATLAB script and this allowed for a detailed analysis of how various parameters of the local proton distribution converged. As an example, we demonstrate in Figure S8 the convergence of the mean value and the standard deviation of the distribution function in the course of the optimization. We found a rapid stabilization of both parameters.

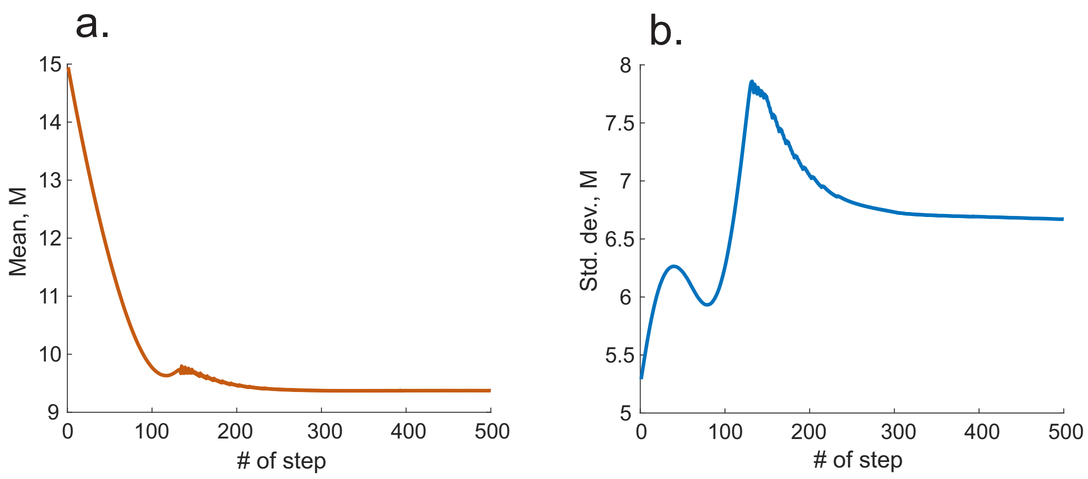

Figure S8: Typical convergence history of (a) mean and (b) standard deviation of the fitted proton density distribution.

Molecular modeling

Modeling of ensembles of spin-labeled BBG chains

The template structure of β-D-glucopyranose was taken from the carbohydrate library. The hydroxyl group at C1, and hydrogens in hydroxy groups at C3 or C4 were removed in the corresponding β-(1→3)/β-(1→4) glycosidic bond linkage. Spin-labeled β-glucan monomer units (SL-monomer) were taken from the canonic rotamer library of the predominant product, substituted at the C6 carbon.[1,5] The positions of SL-monomers were randomly chosen in the polysaccharide chain, excluding β-(1→3) linkage.[5] The fine structure of the barley β-glucan was simulated by the semi-random pattern: for each polysaccharide chain the random pattern of cellotriosyl (DP3) and cellotetraosyl (DP4) blocks with a ratio of $N_{DP3}/N_{DP4} = 3$ was generated;[6,7] the blocks with higher cellulose-like structures (DP5-DP12) were excluded from the simulation due their low fraction. The glycosidic dihedral angles were defined according to IUPAC,[8] and Monte Carlo sampled on the simplified potential energy surface estimated from Ref.;[7] dihedral angles before and after SL-monomers were allowed to be twice as large as for β-D-glucopyranose monomers. An ensemble of 2000 chains with a degree of polymerization of 156 was generated, the polymer chain labeling efficiency was 4.8 (Figure

S9), and the molecular weight varied between 28.7-32.1 kDa. These parameters were used because they were measured for the BBG material used in this work.

Calibration simulations

To relate the standard deviations σ of the electron spin hyperfine shift distributions computed in the ih-RIDME experiments and in the MC modelling to the corresponding standard deviations in the case of homogeneous protons distribution around the electron spin, auxiliary computations were performed. In this series of computations both spin probes and protons were uniformly distributed in a $500 \times 500 \times 500$ nm^3 box with the bulk proton concentration varied within 0.05-8 mol L^{-1}. At a given homogeneous proton density, for each electron spin, the standard deviation σ of the hyperfine shift distribution was taken, as described in the next section, and the σ value averaged over all electron spins was assigned to this particular homogeneous proton density. The resulting calibration curve, which relates σ values to the corresponding homogeneous proton densities, was then used in the BBG ih-RIDME data analysis and in the molecular-modelling-based computations to relate the σ values to the effective local proton density. As explained in the main text, the σ values thus give the original x-axis in such computations, whereas the transformation to effective local proton density is only used for a more intuitive interpretation of the data. The relation between the two scales can be seen in Figure 1f in the main text.

Analysis of modelling results

The electron-proton distance distribution functions ($P(r)$) were calculated for individual conformations of the (spin-labeled) BBG chain as well as for the whole ensemble of spin-labeled BBG chains. Figure S9a represents the distribution of the number of spin-labeled sites per one BBG chain in the generated MC ensemble. Figure S9b shows the distribution of distances from nearby protons to the electron spin position in the spin label for the following cases: (i) just the protons from the spin label moiety itself; (ii) an example of the

most extended BBG conformation with the nearby protons originating only from the nearby sugar moieties in the chain; (iii) an example of the most compact BBG conformation with part of the nearby protons originating from sugar moieties with positions in the BBG chain that are remote from the labeled site; (iv) the electron spin-proton distances distribution computed over the entire ensemble of BBG conformations.

For each $P(r)$ the σ value was calculated using the minimum and maximum distance cut-off as described in Ref.,[4] the resulting range for calculation of the σ value was 1.2-3 nm. The lower cut-off value is associated with the so-called blocking radius.[4] The choice of its value in this work is motivated by the evaluation of the typical distance between the closest protons in the BBG chain. Those are geminal protons of CH_2-group ($r_{\mathrm{H-H}} = 0.18$ nm) and axial protons in sugar pyranose rings ($r_{\mathrm{H-H}} = 0.26 - 0.30$ nm). The flip-flop transition if the group of former protons is faster, however, less critical for the rate of observable spectral diffusion. Therefore, we took into account the second type of proton pairs. Since the distance between them is ca. 2 times larger than that of protons in a water molecule (0.15 nm), we have correspondingly increased the blocking radius by 2 times. The maximum electron-proton cut-off distance was selected to ensure the convergence: further increase of the maximum distance did not result in any significant changes in computed σ values.

For a particular spin-labeled site and a particular BBG chain conformation the σ value was computed as follows. First, distances from the electron spin localization point to all protons within the electron-proton distance range 1.2-3.0 nm were computed. Second, the standard deviations of the hyperfine shifts for all protons were summed according to the formula:

$$\sigma_j^2 = \sum_{i=1}^{N_j} \sigma_{ij}^2 = \sum_{i=1}^{N_j} \frac{0.0555\,\mathrm{MHz}^2 \cdot \mathrm{nm}^6}{r_{ij}^6}. \tag{S4}$$

Here, j is the index of the electron spin, i is the index of one of the N_j protons within the 1.2-3.0 nm range around the j-th electron spin, and r_{ij} is the corresponding electron spin-proton distance. The σ values in the case of homogeneous spin labels and protons distribution in a box were computed in the same way.

The C_{H} distribution for spin labels in BBG was evaluated by comparison of the σ values with homogeneous calibration samples. The resulting distribution can be seen as a histogram in the upper panel of Figure 3a. This distribution was well-fitted by a Gaussian distribution function with the mean value of 4.88 $\mathrm{mol\,L^{-1}}$ and a width of 1.45 $\mathrm{mol\,L^{-1}}$. Two BBG chain conformers with substantially different estimated local proton densities (2.1, respectively, 9.1 $\mathrm{mol\,L^{-1}}$) were visualized (Figure 3a and Figure S10).

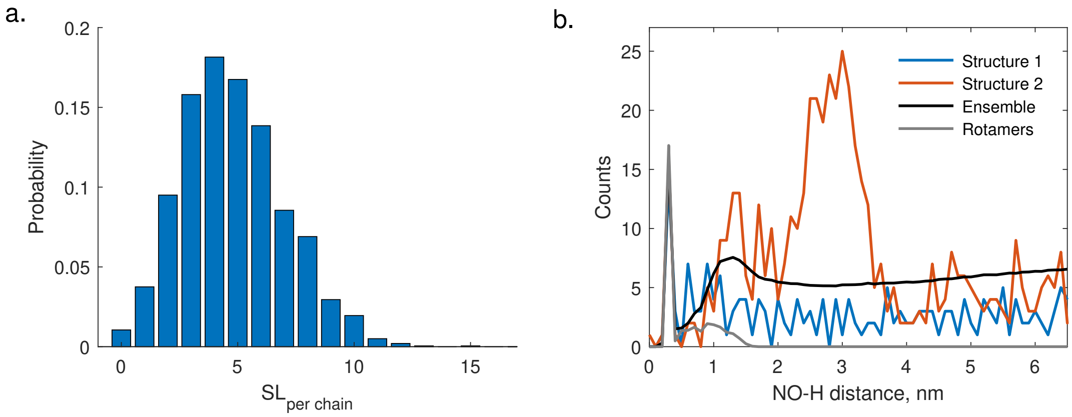

Figure S9: (a) The probability of the spin labels number in the BBG chain for the generated ensemble. (b) The $P(r)$ for structure 1 ($C_{\mathrm{H}} = 2.1$ M) and structure 2 ($C_{\mathrm{H}} = 9.1$ M), averaged over the ensemble and averaged over all SL rotamers.

a.

b.

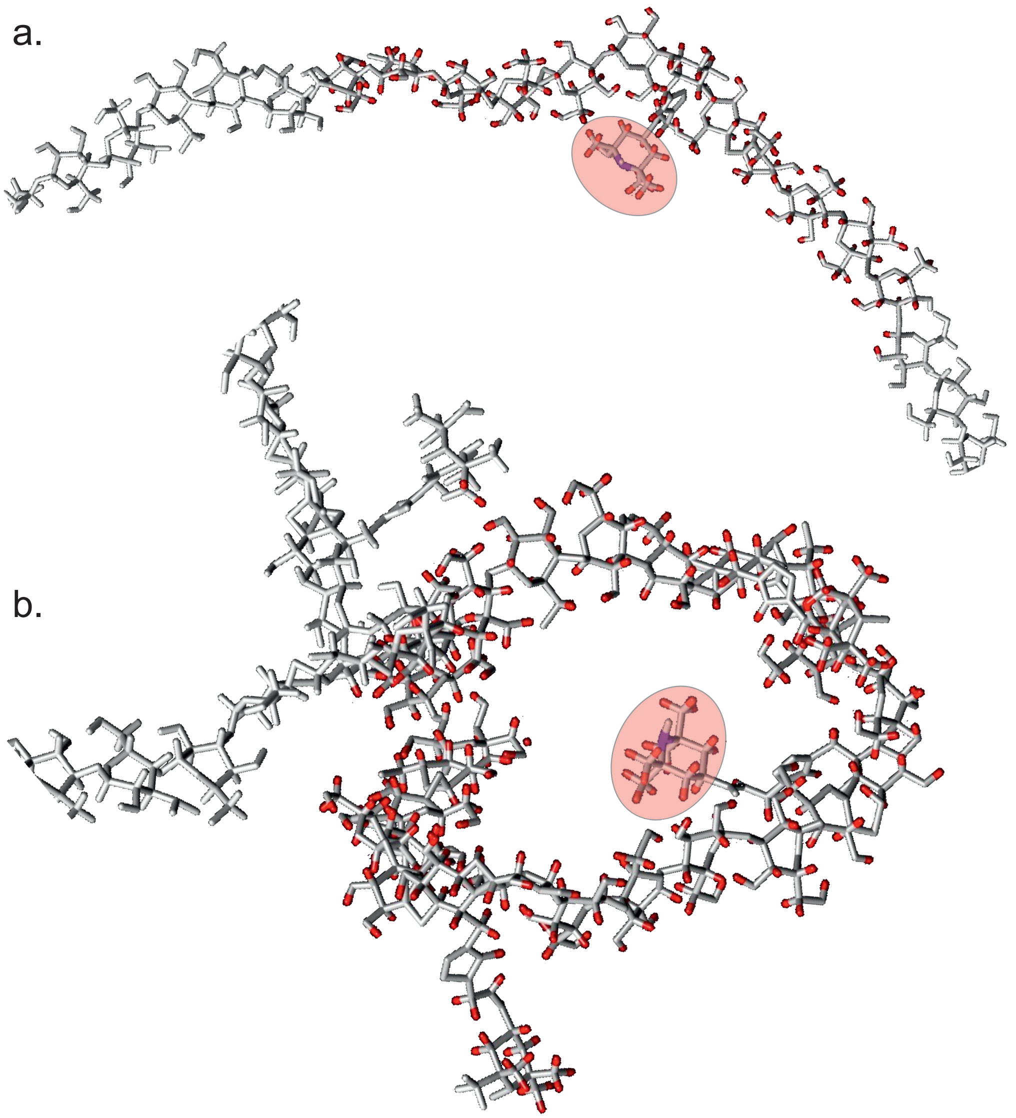

Figure S10: Scaled-up versions of the conformers shown in Figure 3b. The spin label fragment is highlighted in pink. Red atoms are protons within the 2.5 nm sphere around the spin label.

References

(1) Wu, X.; Boulos, S.; Yulikov, M.; Nyström, L. Site-Selective and Stochastic Spin Labelling of Neutral Water-Soluble Dietary Fibers Optimized for Electron Paramagnetic Resonance Spectroscopy. *Carbohydr. Polym.* **2022**, *293*, 119724.

(2) Tschaggelar, R.; Kasumaj, B.; Santangelo, M. G.; Forrer, J.; Leger, P.; Dube, H.; Diederich, F.; Harmer, J.; Schuhmann, R.; García-Rubio, I.; Jeschke, G. Cryogenic 35GHz Pulse ENDOR Probehead Accommodating Large Sample Sizes: Performance and Applications. *J. Magn. Reson.* **2009**, *200*, 81–87.

(3) Keller, K.; Doll, A.; Qi, M.; Godt, A.; Jeschke, G.; Yulikov, M. Averaging of Nuclear Modulation Artefacts in RIDME Experiments. *J. Magn. Reson.* **2016**, *272*, 108–113.

(4) Kuzin, S.; Jeschke, G.; Yulikov, M. Diffusion Equation for the Longitudinal Spectral Diffusion: the Case of the RIDME Experiment. *Phys. Chem. Chem. Phys.* **2022**, *24*, 23517–23531.

(5) Syryamina, V.; Wu, X.; Boulos, S.; Nyström, L.; Yulikov, M. Pulse EPR Spectroscopy and Molecular Modeling Reveal the Origins of the Local Heterogeneity of Dietary Fibers. *Carbohydr. Polym.* **2023**, *319*, 121167.

(6) Staudte, R.; Woodward, J. R.; Fincher, G. B.; Stone, B. A. Water-Soluble (1→ 3),(1→ 4)-β-D-Glucans from Barley (Hordeum Vulgare) Endosperm. III. Distribution of Cellotriosyl and Cellotetraosyl Residues. *Carbohydr. Polym.* **1983**, *3*, 299–312.

(7) Li, W.; Cui, S. W.; Wang, Q.; Yada, R. Y. Study of Conformational Properties of 1024 Cereal β-Glucans by Computer Modeling. *Food Hydrocoll.* **2012**, *26*, 377–382.

(8) IUPAC-IUB Joint Commission on Biochemical Nomenclature. Nomenclature of Carbohydrates (Recommendations 1996), *Carbohydr. Res.* **1997**,
