# Supplementary material for: Quantification of Distributions of Local Proton Concentrations in Heterogeneous Soft Matter and Non-Anfinsen Biomacromolecules
Source: J Phys Chem Lett. 2024 May 17;15(21):5625–32. doi: 10.1021/acs.jpclett.4c00825 (PMC11145652; doi:10.1021/acs.jpclett.4c00825)
Supplement: Supplementary file 2 — jz4c00825_si_002.pdf [file jz4c00825_si_002.pdf]

jz-2024-008255.R1

Name: Peer Review Information for "Quantification of Inhomogeneous Local Proton Density Distributions in Soft Matter, Unstructured Macromolecules and Non-Anfinsen Biomacromolecules"

First Round of Reviewer Comments

Reviewer: 1

Comments to the Author

This is a new approach in quantification of proton density surrounding spin labels as demonstrated in polysaccharides, which is applicable for biomarcomolecules. Overall, the data were well qualified, and nicely presented. However, I suggest the authors to consider following comments.

1) It would be nice to clarify the parameters as shown in Tables and figure captions, and the current version is difficult to follow.

2) The impact of distance between spin labels in polysaccharides on the quantification as illustrated in the approach has to be discussed or commented in the manuscript.

Reviewer: 2

Comments to the Author

In this letter, the authors test and prove the ability to measure the local proton concentration around a paramagnetic center. The work relies on two pulse sequences, dubbed ih-RIDME and hf-DEER. The experimental work and analysis are very robust and there is no concerns in my opinion. However, the article is difficult to read, and I suggest a major revision to be understood by a broader audience.

The introduction is very long compared to the experimental section. I suggest shortening the introduction, which represents ~ 5 pages (>1/3 of the letter), and instead expand the experimental/analysis further. The figures should be edited to make them more reader friendly.

The discussion of the hf-DEER is somewhat superficial, and this is a bit disappointing. The link between the ih-RIDME and benefits of the hf-DEER may be clear for an expert, however it should be expanded to help non-experts “see” how this could be impactful.

This includes explaining how one would extract different radical distances for different CH concentration. This part brings a lot of value to the letter by the potential applications.

Details:

Why divide by short Tmix? Is it to remove additional decay contribution? This is not well explained.

The figure 1 should be split or simplified to ease the reading of the article. For instance, the “raw ih-RIDME” figure 1d may be placed in the SI. If this is not critical information

Figure 1.f is unclear, what do you mean by “high, mid and low”. This is not discussed in the text. My guess is that you refer at the proton concentration and the contribution of each of the environment showed in the CH distribution (figure 1g), but I’d like to be sure and see a link with the distribution.

L40 to 56 p7: those two important sentences are very convoluted, though important.

L29 p 8, why discuss figure 2c now? The order of the figure referencing is a bit unclear. This should be in figure 1, not figure 2 or I am missing the point.

L10-20 p 10: you could show a 2D DEER for different Tmix? The idea of combining DEER and hf is very neat but it is not sufficiently developed here. This leads to a very dry discussion about that possibility and at the same time reduces the impact of the ih-RIDME.

Figure 2c is very convoluted. I have a hard time to really follow the flow of this figure. If I am not mistaken, you do not show in the SI the local concentration obtained by MD, and instead discuss the electron-proton distance distribution functions. They are related by not a direct comparison.

L17 p 14: could you explain how useful it could be for DNP? This comes at the very end but if it’s relevant, then it should be in the discussion.

Reviewer: 3

Comments to the Author

This is a very novel advance in the characterization of macromolecular structure that is applicable to both monodisperse and polydisperse structure and conformations. That is, from proteins of identical structure to heavily modified glycoproteins with poly-disperse molecular weights, and even to synthetic polymers with a broad range of molecular weights and conformations. It appears to provide the same information as small angle scattering methods but with totally different requirements on samples, sample preparation, and instrumentation. It appears to involve several novel aspects of widely used magnetic resonance methods for macromolecular structure characterization that are well thought out and cleverly applied.

This combination of novel approach and wide applicability in a wide range of chemical and materials research areas makes it a very appropriate and impactful topic for JPCLetts. Unfortunately, the present article is written for someone very knowledgeable in the specialized field of RIDME spectroscopy and its applications. This paper needs revision to make it readily understandable to the broad range of physical chemists.

In particular, the paper should:

1) Explain briefly the physical concept behind the ability of this RIDME method to detect the presence and concentration of protons near a spin label.

2) Relate the concentration information obtained from this method to the information obtained from other methods such as small angle scattering. Is there additional or complementary information or simply a better route to the same information?

3) Explain the different types of information that can be obtained with this method if random labelling versus site directed labelling is used.

The general reader should not need to know all the technical details and nuances of the method but should be given insights into the underlying principles and the relation to other methods of obtaining similar information.

Another shortcoming of the present version of the paper concerns the accuracy of results. There is really no comparison or validation of the concentration profile obtained. There is an MD calculation which could be used as a partial validation of results, but aside from a vague assurance that the results are good, there is little for the reader to see and evaluate. The experimental concentration

profile in Fig 2C obtained from RIDME should be compared with the profile obtained from MD calculations. The caption for Fig. 2C is an inadequate explanation of what is shown in the upper pane. It would be very desirable to compare the distance distribution obtained from the MD with a distance distribution simulated from a SANS or SAXS measurement based on the MD with one derived from the RIDME measurements. This would help many readers place the RIDME in context with other approaches described in the introduction to this paper AND it would allow readers to evaluate the accuracy and performance.

I am somewhat uncomfortable with the use of three Gaussian distributions to analyze the results from an ensemble of unstructured macromolecules. That is, 9 independent parameters from what I would expect to be a rather smooth distribution. It strongly suggests to me a significant degree of overfitting (and is another reason for comparing a distribution obtained from the MD calculations.) There are many simple distributions commonly used in statistical description of distributions that have the same general shape but far fewer independent parameters. For instance: log-normal, gamma, F, etc. And there are statistical methods for comparing distributions that would be more objective than the plots in Fig 1g,i.

Some revision of this paper is required to convert it from a paper accessible only to a small group of specialized EPR researchers into a paper of interest to a broad range of physical chemists interested in macromolecular structure and dynamics.

Author's Response to Peer Review Comments:

Dear Editor,

we submit a revised version of our manuscript for your consideration. The response to the reviewer comments can be found in the attached PDF file.

With best regards,

on behalf of all authors,

Dr. Maxim Yulikov

ETH Zurich, 20.04.2024

RESPONSE TO REVIEWER REPORTS

We would like to thank the reviewers for their effort to read and evaluate our manuscript, for the generally positive judgements and for providing valuable critical comments.

***Reviewer 1** comments:*

This is a new approach in quantification of proton density surrounding spin labels as demonstrated in polysaccharides, which is applicable for biomarcomolecules. Overall, the data were well qualified, and nicely presented. However, I suggest the authors to consider following comments.

1) It would be nice to clarify the parameters as shown in Tables and figure captions, and the current version is difficult to follow.

Reply: Table and figure captions as well as their discussion in the text have been reconsidered and revised where appropriate.

2) The impact of distance between spin labels in polysaccharides on the quantification as illustrated in the approach has to be discussed or commented in the manuscript.

Reply: We extended the discussion of the 2D correlation experiment, according to the suggestions of this and two other reviewers.

***Reviewer 2** comments:*

In this letter, the authors test and prove the ability to measure the local proton concentration around a paramagnetic center. The work relies on two pulse sequences, dubbed ih-RIDME and hf-DEER. The experimental work and analysis are very robust and there is no concerns in my opinion. However, the article is difficult to read, and I suggest a major revision to be understood by a broader audience.

The introduction is very long compared to the experimental section. I suggest shortening the introduction, which represents ~ 5 pages (>1/3 of the letter), and instead expand the experimental/analysis further. The figures should be edited to make them more reader friendly.

Reply: Just the length of the introduction itself is not yet a valid argument to shorten it. We intentionally wrote the introduction in this way to introduce a number of necessary concepts, which, we believe, will help the broad readership to see where to place this new method in the context of structural studies. However, we partially accept the criticism of the reviewer, in relation to the first few paragraphs of the introduction, which serve more as a short "historical overview". This first part of the introduction was further shortened, but due to the need to add few explanations regarding the DNP and the RIDME, as requested by reviewers, we after all could not really shorten introduction. More explanations, in line with the reviewers' requests were added in the results and discussion part of the manuscript.

The discussion of the hf-DEER is somewhat superficial, and this is a bit disappointing. The link between the ih-RIDME and benefits of the hf-DEER may be clear for an expert, however it should be expanded to help non-experts "see" how this could be impactful. This includes explaining how one would extract different radical distances for different CH concentration. This part brings a lot of value to the letter by the potential applications.

Reply: We would like to emphasize that the design of a fitting protocol and an in-depth hf-DEER data analysis probably has to wait for a spin-labelled protein study, where one can analyse an intramolecular distance distribution between two specific labelling sites in correlation to local proton density. In the revision of the present manuscript, we extended the general discussion of this 2D correlation experiment in the corresponding paragraphs (see new pages 13-15).

The advantage of the polysaccharide test system was that all paramagnetic sites are surrounded with the same type of protons, which simplified the proof of principle determination of the distribution of local proton densities. The stochastic labelling of BBG samples, however, makes it impossible to determine independently the distribution of local electron spin concentrations, which is mandatory for a 2D correlation analysis. On such stochastically labelled systems the full quantitative analysis of DEER and ih-RIDME correlations is probably only possible along with a detailed molecular modelling, including modelling of aggregates. Such a computation is however a challenge on its own, and it goes much beyond the scope of the present report.

Details:
Why divide by short Tmix? Is it to remove additional decay contribution? This is not well explained.

Reply: The reason for this division is the need to remove the RIDME-related artefacts. This explanation with reference is now added in the text.

The figure 1 should be split or simplified to ease the reading of the article. For instance, the "raw ih-RIDME" figure 1d may be placed in the SI. If this is not critical information

Reply: We rather consider this as critical information: the first submission version figure 1d (figure 2a in the revised manuscript) shows the difference between the raw data for free BBG and for BBG with two different amounts of added CalW. This emphasizes the fact that the change of the local proton densities between these samples is visible directly in the experimental data prior to any data processing. Figure 1e (figure 2b in the revised manuscript) shows the example of divided traces ready to be fitted for the same three samples. We have rearranged the panels to make a manuscript with three figures instead of two. The figure captions were revised and extended to improve clarity.

Figure 1.f is unclear, what do you mean by "high, mid and low". This is not discussed in the text. My guess is that you refer at the proton concentration and the contribution of each of the environment showed in the CH distribution (figure 1g), but I'd like to be sure and see a link with the distribution.

Reply: We added reference concentration values, corresponding to the 1/e decay at these time points. This way, there is now a quantitative measure for the three concentration ranges. The corresponding discussion in the manuscript text has been revised and extended.

L40 to 56 p7: those two important sentences are very convoluted, though important.

Reply: We extended and simplified this paragraph.

L29 p 8, why discuss figure 2c now? The order of the figure referencing is a bit unclear. This should be in figure 1, not figure 2 or I am missing the point.

Reply: We have now rearranged the order of discussing experimental data in the revised manuscript and sorted the figure subplots accordingly, to avoid any possible confusion.

L10-20 p 10: you could show a 2D DEER for different Tmix? The idea of combining DEER and hf is very neat but it is not sufficiently developed here. This leads to a very dry discussion about that possibility and at the same time reduces the impact of the ih-RIDME.

Reply: No, unfortunately not for these samples. This has been briefly explained in the original version of the manuscript. Now we substantially extended this explanation, see new pages 13-15. Here, we only claim priority for the idea of correlating distances and proton densities by proposing the 2D experiment and demonstrating that the measurements work fine and the correlation does exist and is clearly detectable and interpretable at the qualitative level. Full analysis will require site-directed spin labelling of protein or nucleic acid molecules. This work is in progress in our labs.
We would like to point out that in our opinion, already the 1D ih-RIDME analysis of heterogeneous samples is a significant spectroscopic advance, and it does not necessarily require additional support to justify the publication.

Figure 2c is very convoluted. I have a hard time to really follow the flow of this figure. If I am not mistaken, you do not show in the SI the local concentration obtained by MD, and instead discuss the electron-proton distance distribution functions. They are related by not a direct comparison.

Reply: The original Figure 2c is now split into three subplots – Figure 1f and Figure 3a,b. The explanations related to these two subplots have been substantially extended.

L17 p 14: could you explain how useful it could be for DNP? This comes at the very end but if it's relevant, then it should be in the discussion.

Reply: The discussion of DNP is now extended both in the Introduction block and in the new paragraph at the end of the Conclusions block.

***Reviewer 3** comments:*

This is a very novel advance in the characterization of macromolecular structure that is applicable to both monodisperse and polydisperse structure and conformations. That is, from proteins of identical structure to heavily modified glycoproteins with poly-disperse molecular weights, and even to synthetic polymers with a broad range of molecular weights and conformations. It appears to provide the same information as small angle scattering methods but with totally different requirements on samples, sample preparation, and instrumentation.

Reply: In fact, the information is not quite the same as in SAXS. In particular, the polysaccharide samples studied here possess both large-scale and small-scale heterogeneity. The large-scale heterogeneity originates from the chain lengths distribution and from the presence of aggregates. The small-scale heterogeneity appears then as differences of the local proton density in the near vicinity of paramagnetically labelled sugar

moieties. SAXS would be a good method if one had a monodisperse non-aggregated polysaccharide solution, or alternatively a macroscopically homogeneous (but microscopically non-homogeneous) sample, such as e.g. polymer melt with local fluctuations of density. The “two-scales heterogeneous” samples studied here represent a very hard case for SAXS. A somewhat rephrased version of this explanation is added in the revised manuscript.

It appears to involve several novel aspects of widely used magnetic resonance methods for macromolecular structure characterization that are well thought out and cleverly applied. This combination of novel approach and wide applicability in a wide range of chemical and materials research areas makes it a very appropriate and impactful topic for JPCLetts.

Unfortunately, the present article is written for someone very knowledgeable in the specialized field of RIDME spectroscopy and its applications. This paper needs revision to make it readily understandable to the broad range of physical chemists.

Reply: As also mentioned in the reply to the reviewer 2 comments, we thoroughly revised the manuscript to improve the broader-audience appeal. The story line was also somewhat rearranged and “straightened” to elevate some potential confusions indicated by the reviewers.

In particular, the paper should:
1) Explain briefly the physical concept behind the ability of this RIDME method to detect the presence and concentration of protons near a spin label.

Reply: We acknowledge that the already existing explanation (last paragraph on page 6 in the original version) is not easy to understand for a broad audience. Hence, we introduced a new paragraph explaining how the local proton density distribution comes about. This explanation is further aided by a visual scheme added to the new Figure 1d.

2) Relate the concentration information obtained from this method to the information obtained from other methods such as small angle scattering. Is there additional or complementary information or simply a better route to the same information?

Reply: As described above, there are issues with analysing SAXS data on such samples, which have chain length distribution, substantial fraction of aggregates and, at the same time, heterogeneity of local site proton density. Therefore, unfortunately, we cannot add such a comparison. We have, however, added in the revised manuscript a discussion of the SAXS limitations for such systems:
“The presented here distributions of local proton density computed from ih-RIDME data have some similarities to the output of the small angle x-ray or neutron scattering experiments (SAXS/SANS). There are, however, important differences between these two techniques. The data provided by ih-RIDME technique are strictly local and do not depend on the larger scale heterogeneity of the sample. In particular, here we can study polysaccharide samples with distribution of chain lengths and with a substantial fraction of aggregates. Also, the ih-RIDME experiment does not set any conditions on the distances between different polymer chains, and thus can be performed in a broad polymer concentrations range, including partial overlap concentrations. In contrast to ih-RIDME, SAXS or SANS experiments work well at two limiting types of conditions: Either one needs to ensure large interchain distances, significantly longer than the average chain length, or one needs to have dense near-homogeneous polymer melt. In the former case, one can potentially determine the conformational ensemble for an isolated polymer chain, under a condition that the chain length distribution is accurately determined, or, better, the sample is monodisperse. In the latter case, one can study local polymer density fluctuations in the melt.
The studied here BBG samples are characterized by both ”large-scale” and ”small-scale” heterogeneity. The large-scale heterogeneity originates from the chain lengths distribution and from the presence of aggregates. The small-scale heterogeneity appears then as differences of the local sugar protons density in the near vicinity of paramagnetically labelled sugar moieties. Such “two-scales heterogeneous” samples represent a very hard case for SAXS/SANS.”

3) Explain the different types of information that can be obtained with this method if random labelling versus site directed labelling is used. The general reader should not need to know all the technical details and nuances of the method but should be given insights into the underlying principles and the relation to other methods of obtaining similar information.

Reply: This discussion is added in the revised manuscript (see pages 13-15), at the point where we discuss the 2D RIDME-DEER correlation experiment.

Another shortcoming of the present version of the paper concerns the accuracy of results. There is really no comparison or validation of the concentration profile obtained. There is an MD calculation which could be used as a partial validation of results, but aside from a vague assurance that the results are good, there is little for the reader to see and evaluate. The experimental concentration profile in Fig 2C obtained from RIDME should be compared with the profile obtained from MD calculations. The caption for Fig. 2C is an inadequate explanation of what is shown in the upper pane. It would be very desirable to compare the distance distribution obtained from the MD with a distance distribution simulated from a SANS or SAXS measurement based on the MD with one derived from the RIDME measurements. This would help many readers place the RIDME in context

with other approaches described in the introduction to this paper AND it would allow readers to evaluate the accuracy and performance.

Reply: We explained above why SAXS/SANS is not a good comparison method for our case. The Monte-Carlo-based conformational ensemble of non-aggregated BBG chains has been compared to the very-low-concentration DEER data in our previous paper (Syryamina *et al*. Carbohydrate Polymers 2023). Those experiments confirm that the conformational distribution predicted by MC is realistic. The upper pane of the Figure 2C (Figure 3a in the revised manuscript) shows the comparison of the effective local proton concentrations according to the MC ensemble and the shape of the low concentration peak in the ih-RIDME data. Please note that only the low-concentration peak corresponds to the non-aggregated BBG chains, the two other peaks we attribute to the aggregates, which were not modelled in the MC computations. We revised figure caption and extended the corresponding discussions in the manuscript text.

I am somewhat uncomfortable with the use of three Gaussian distributions to analyze the results from an ensemble of unstructured macromolecules. That is, 9 independent parameters from what I would expect to be a rather smooth distribution. It strongly suggests to me a significant degree of overfitting (and is another reason for comparing a distribution obtained from the MD calculations.) There are many simple distributions commonly used in statistical description of distributions that have the same general shape but far fewer independent parameters. For instance: log-normal, gamma, F, etc. And there are statistical methods for comparing distributions that would be more objective than the plots in Fig 1g,i.

Reply: This is likely a misunderstanding. First, the ih-RIDME data were fitted with a model-free distribution of local proton densities, and only then this model-free distribution was approximated as a sum of three Gaussian peaks. For the model-free fit stability please refer to the ESI Figure S7. Second, only one of the three peaks in the local proton density distribution belongs to the non-aggregated BBG chains, the two others reflect presence of dense and less dense aggregates. Therefore, the three peaks are of different nature and should not be described by a single function, in our opinion. The presence of aggregates has also been demonstrated by the concentration-dependent DEER experiments in our recent publication (Syryamina *et al*. Carbohydrate Polymers 2023). Furthermore, the size-exclusion chromatography data clearly indicate aggregates in these samples (*ibid*). We further emphasized these points in the revised manuscript.

Some revision of this paper is required to convert it from a paper accessible only to a small group of specialized EPR researchers into a paper of interest to a broad range of physical chemists interested in macromolecular structure and dynamics.

Reply: Complex problems may necessitate a description that requires considerable effort by the reader. Yet we hope that our explanations and the changes made in the revised manuscript resolve at least the main concerns of this and other reviewers.

jz-2024-008255.R2

Name: Peer Review Information for "Quantification of Distributions of Local Proton Concentrations in Heterogeneous Soft Matter and non-Anfinsen Biomacromolecules"

Second Round of Reviewer Comments

Reviewer: 3

Comments to the Author

The authors seem to have a very different concept of the paper and its importance than I do. I feel that the new method is a very significant and important advance in the characterization of macromolecular structure and dynamics, of nanostructures, and of phase separation. I think that this method will be of extremely strong interest and impact for a broad range of scientists ranging from structural and functional biology/biochemistry to soft matter, and nanomaterials. They all face challenges in characterizing structure and dynamics with current methods and they need better tools for doing so. The technique described in this paper is potentially very important addition, for example, to small angle scattering methods. This broad community already knows the limitations of current methods and the need for new approaches, like the RIDME described here. They need a basic introduction to the principles behind this method, the information obtainable from it, and a how its information complements information from techniques such as SAXS/SANS.

Unfortunately, this paper seems to be written for a very limited audience of researchers actively involved in RIDME and DEER spectroscopy. Most of the first four pages of the paper seem to be a textbook justification of why RIDME spectroscopists ought to be concerned about unstructured and poorly structured materials at all. And the paper ends with a similar justification for relevance in DNP research. Then, instead of briefly presenting the concepts behind the technique and perhaps contrasting them with small angle scattering approaches, there is a detailed discussion of several pulse sequences and variations of the RIDME and DEER experiments that will only be understandable to a limited number of readers already very familiar with these methods.

I feel that one major significance of this method is that it provides information that complements, but goes beyond, what is currently available from other techniques. But this paper focuses more on the lenient sample requirements than on the new kinds of information obtained. The detailed response to the first review notes a paper published last year that already presented results using the method being introduced here and that shows the validity of the experimental results. This prior publication would seem to eliminate the need for the detailed description of the data analysis procedure in this submission and allow more discussion of how this technique fits in with existing ones for structural characterization of heterogeneous and unstructured materials and biomaterials.

The authors choice of what is important enough to include in the paper and what isn't seems strange. For instance, the need to define Euler's constant 'e' in JPCL.

In its present form, this manuscript will probably take some time to reach its full impact because its significance will initially be confined largely to the RIDME/DEER community.

Reviewer: 2

Comments to the Author

Quantification of Inhomogeneous Local Proton Density Distributions in Soft Matter, Unstructured Macromolecules and non-Anfinsen Biomacromolecules

The authors have addressed some of my comment and rebuked others. I think the article is ok. As previously written, I find the science to be good and well executed. However, I still find reading this letter to be tedious and this will impact the interest in the community. This is not in our interest; the work is good and must be read.

While it’s a tedious process, the introduction is too long for a letter. It boils down to four points.

1- Flexible polymers do not have efficient way to probe the coiling/folding.

2- Biomolecules such as intrinsically disordered proteins are flexible biopolymer

3- Pulse EPR pulse sequence RIDME is sensitive to local proton concentration.

4- We use RIDME to probe coiling/folding of biolpolymers dissolved in deuterated media. (this is key in my opinion).

Based on these four points, the introduction could be much shorter and the message clearer. Often you need to go to the point faster and not dwell on details/comments.

Minor points and the list is not exhaustive.

P3: “In general, we have much better-developed characterization tools to determine the structure of the ordered parts of macromolecules, but, at the same time, we are substantially more limited in the selection of methods to determine the details of the broad conformational distributions of the corresponding unstructured parts of the macromolecules of interest.”

In general we have a vast toolbox to characterize the structured segments of macromolecules but have fewer tools to discern the diverse conformational states of their unstructured counterparts.

L49 p3: I think you miss “when”?

are particularly well suited “when” combined with characterization by NMR and Raman.

L21, p4: “While such macromolecules are not involved as actors in the intracellular biochemistry, they

are involved in cellular signaling and in energy storage of the living organisms besides their

structural function in cell walls and glycocalyx”

“Although not directly engaged in intracellular biochemistry, these macromolecules are involved in the cellular signaling, the energy storage, and the structural support within cell walls and glycocalyx.”

Note that if they are involved in the signaling, they are part of the biochemistry.

L25 p4, “Polysaccharides are also seen as an important constituent of human diet. DFs do affect our nutritional biochemistry and hence our health and well-being.”

This may be common knowledge, but a reference is needed.

L55. P4 “Importantly for our current topic, it has been already established that the different local proton distributions for different spin label conformations can lead to delay-time-dependent variations of the electron spin-spin distance distributions even in well-structured biopolymers.”

I don’t understand what you mean. Is this what you mean?

“Importantly, it has been already established that different spin label conformations, and their associated local proton concentration, around can affect the outcome electron spin-spin distance measurements.”

L33 p5, “These interactions include electrostatic contributions and hydrophobic effects, they

depend on steric features of particular chains and small molecules, as well as on the hydration of the specific moieties. Such interactions affect the conformational distributions, and also determine the propensity for agglomeration, gelation or liquid-liquid phase separation (LLPS) phenomena”

Do you mean “These interactions, e.g. electrostatic, hydrophobic effects, or hydration, depend on the steric features of the chains and small molecules. They affect the conformational distributions, and determine the propensity to agglomerate, form gels or make liquid-liquid phase separation (LLPS) phenomena

L49 p5 “This characteristic of DFs is important in relation to their role in human nutrition.”

Can you add a ref?

L11 p6 In ref 46, my recollection is that the protons on the biradical are important, is RIDME sensitive to this? The first author on ref 46 has a recent publication in JPCL that somewhat contradicts the point developed.

Overall, I doubt RIDME is helpful for biradical design but, it this is important, your point is actually very relevant when one wants to understand if a biradical has an affinity for a surface in DNP. I think you should consider this instead.

L31 p6. “quite well-know” is unnecessary, it’s well-known within the EPR community only.

“based on a the relaxation-induced dipolar modulation enhancement pulse EPR technique (RIDME). The technique was originally designed to measure electron spin-spin interactions,48,49 as a possible alternative to the commonly used double electron-electron resonance (DEER) technique.”

L41 p6. “can be quite reliably and accurately” isn’t it the same?

L30, p7. “At the same time, RIDME was found to characterise the structure of the local protonic environment of a spin label.”

This redundant

L23, p8. Typo at “deviation”

L37, p8 “In this work we always recorded five traces, and an additional reference

trace with short mixing time.”

This is not five, but six traces.

L5, p9, where does D/sigma^3 appears in the equation? Please clarify.

L55, p9, “of course” is not needed.

L49, p10. “In particular, there are indications that the width of the component appearing at the highest local proton concentration might be unstable”

Do you mean “In particular, it seems that highest local concentration distribution is unstable” ?

L29, p11 “Overall, these experiments indicate that the ih-RIDME

data fitting can help following the interchain contacts statistics with good resolution. In our

opinion, this is a very valuable result, as such contacts statistics is one of the key questions to

ask when one wants to describe weak aggregates, including LLPS phenomena in biopolymers

studies.”

Do you mean “Overall, these experiments demonstrate that the ih-RIDME data fitting can track interchain contact statistics with high resolution. Us understanding such contact statistics is crucial for characterizing weak aggregates, including phenomena like LLPS, in biopolymer studies, making this outcome is highly valuable”.

L47, p13 “which would go far beyond the scope” may be “which would go beyond the scope”

L18, p20 “We anticipate that ih-RIDME and hf-DEER techniques might attract significant attention in the DNP research, because these techniques aim precisely at filling this gap of information on the local proton distributions around paramagnetic centers.”

I think you have a point, RIDME could help understand radical affinity with sample. This is something that would be good for DNP. When it comes to understand radicals, I could oppose the following arguments:

- can the ZQ e-e cross-relaxation contaminate the RIDME decay? If yes, how do you remove it?

- In DNP glass forming solution, would we see a difference between biradicals? And what would you understand from it? In general, biradicals are made to be as soluble as possible to avoid aggregation. To probe aggregation, a DEER decay would prove more useful?

- The ENDOR spectra may be in general more useful as Ref 46 shows protons with large hyperfine couplings are important.

- Can MIMS ENDOR be helpful in that case, the intensity of the central ENDOR peak correlates with local proton concentration?

Author's Response to Peer Review Comments:

Dear Editor,

we submit for your consideration the second revision of our manuscript, with style adjustments in line with the additional reviewer comments received. Please find our step-by-step response to reviewers attached as a PDF file.

Best regards,

on behalf of all authors,

Maxim Yulikov

ETH Zurich, 13.05.2024

Reply to reviewer comments.

Reviewer 3 Comments:

The authors seem to have a very different concept of the paper and its importance than I do.

Thanks for this remark, which matches our own assessment. To some extent, we believe that the authors define the concept of a paper, which then may or may not be suitable for a given journal. That said, we can see your points, some of which are echoed by Reviewer 2. We tried to find a compromise between our own concept and the concept that you suggest.

I feel that the new method is a very significant and important advance in the characterization of macromolecular structure and dynamics, of nanostructures, and of phase separation. I think that this method will be of extremely strong interest and impact for a broad range of scientists ranging from structural and functional biology/biochemistry to soft matter, and nanomaterials. They all face challenges in characterizing structure and dynamics with current methods and they need better tools for doing so.

We share this opinion. We are grateful for your attempt to make this work more accessible to a broader community.

The technique described in this paper is potentially very important addition, for example, to small angle scattering methods. This broad community already knows the limitations of current methods and the need for new approaches, like the RIDME described here. They need a basic introduction to the principles behind this method, the information obtainable from it, and a how its information complements information from techniques such as SAXS/SANS. Unfortunately, this paper seems to be written for a very limited audience of researchers actively involved in RIDME and DEER spectroscopy. Most of the first four pages of the paper seem to be a textbook justification of why RIDME spectroscopists ought to be concerned about unstructured and poorly structured materials at all. And the paper ends with a similar justification for relevance in DNP research. Then, instead of briefly presenting the concepts behind the technique and perhaps contrasting them with small angle scattering approaches, there is a detailed discussion of several pulse sequences and variations of the RIDME and DEER experiments that will only be understandable to a limited number of readers already very familiar with these methods.

We made an honest attempt to reduce background material in the introduction. Yet, we believe that a section of the introduction on weakly structured materials and biomacromolecules is necessary.

I feel that one major significance of this method is that it provides information that complements, but goes beyond, what is currently available from other techniques.

We agree and now try to stress this point. In the new version, not all of this discussion is in the introductory paragraphs, because some of it requires information that comes up only in the paragraphs on results.

But this paper focuses more on the lenient sample requirements than on the new kinds of information obtained.

We agree that the ratio between the different aspects was not optimal in the first revised version. Yet, the fact that ih-RIDME can deal with more heterogeneous samples is not simply a technical detail or a matter of convenience. Systems that contain dispersed and aggregated/condensed macromolecules under equilibrium or metastable conditions are important and hence it is important to have characterization techniques for them. Therefore, our revised version stresses both aspects, the new kinds of information and the broader range of accessible systems.

The detailed response to the first review notes a paper published last year that already presented results using the method being introduced here and that shows the validity of the experimental results. This prior publication would seem to eliminate the need for the detailed description of the data analysis procedure in this submission and allow more discussion of how this technique fits in with existing ones for structural characterization of heterogeneous and unstructured materials and biomaterials.

Removing part of the description of data analysis would indeed make the manuscript easier to read for non-specialists, but unfortunately our previous PCCP publication only deals with deriving the spectral diffusion equation and applying it to *homogeneous* samples. All the methodology for analysing *heterogeneous* samples is presented and discussed here for the first time and it is heterogeneous samples, where this technique provides information that goes beyond the one from other techniques.

The authors choice of what is important enough to include in the paper and what isn't seems strange. For instance, the need to define Euler's constant 'e' in JPCL.

We removed the definition of Euler’s constant.

In its present form, this manuscript will probably take some time to reach its full impact because its significance will initially be confined largely to the RIDME/DEER community.

We are grateful that you push us to make our work more accessible. We feel that this requires some balance. Essential technical details must be explained somewhere, and a journal devoted to physical chemistry appears to be a suitable outlet for a careful description. Some delay in uptake may be unavoidable due to the novelty of the approach. That said, JPCL certainly aims at a broader audience of physical chemists and in our second revised version we try to discuss information content and application range of ih-RIDME on a more general level. To this end, we also consider its relation to scattering techniques and (sm)-FRET. We have shortened other paragraphs in order to keep the length and complexity of the manuscript in a reasonable range.

Reviewer 2 Comments:

The authors have addressed some of my comment and rebuked others. I think the article is ok. As previously written, I find the science to be good and well executed. However, I still find reading this letter to be tedious and this will impact the interest in the community. This is not in our interest; the work is good and must be read.

This comment reinforces remarks by Reviewer 3 that we discussed above. We thank you, too, for insisting on making this work more accessible. Part of the problem arises from complexity of the work, which requires to discuss a new method, polymer physics aspects, and particulars of the carbohydrate model system. That said, we were certainly too ambitious regarding the amount of background information and regarding future potential of the technique in special fields. By removing or reducing such parts, we have made some space for more general explanations aimed at a broader audience.

While it’s a tedious process, the introduction is too long for a letter. It boils down to four points.

1- Flexible polymers do not have efficient way to probe the coiling/folding. 2- Biomolecules such as intrinsically disordered proteins are flexible biopolymer 3- Pulse EPR pulse sequence RIDME is sensitive to local proton concentration. 4- We use RIDME to probe coiling/folding of biolpolymers dissolved in deuterated media. (this is key in my opinion).

Based on these four points, the introduction could be much shorter and the message clearer. Often you need to go to the point faster and not dwell on details/comments.

We agree that the amount of details and comments was too large. The introduction is still rather long (we need to say something about the model system and making the connection between polymer physics and flexible biomacromolecules is important). However, we think that the new introduction is better suited to get a broader audience interested.

Minor points and the list is not exhaustive.

We have rewritten several sections of the manuscript, taking into account the general sentiment in your comments.

P3: “In general, we have much better-developed characterization tools to determine the structure of the ordered parts of macromolecules, but, at the same time, we are substantially more limited in the selection of methods to determine the details of the broad conformational distributions of the corresponding unstructured parts of the macromolecules of interest.”

In general we have a vast toolbox to characterize the structured segments of macromolecules but have fewer tools to discern the diverse conformational states of their unstructured counterparts.

We accepted this suggestion. The text is modified accordingly.

L49 p3: I think you miss “when”? are particularly well suited “when” combined with characterization by NMR and Raman.

Thanks. This sentence changed during rewriting.

L21, p4: “While such macromolecules are not involved as actors in the intracellular biochemistry, they are involved in cellular signaling and in energy storage of the living organisms besides their structural function in cell walls and glycocalyx” “Although not directly engaged in intracellular biochemistry, these macromolecules are involved in the cellular signaling, the energy storage, and the structural support within cell walls and glycocalyx.”

Note that if they are involved in the signaling, they are part of the biochemistry.

We accepted this suggestion. We modified the text accordingly.

L25 p4, “Polysaccharides are also seen as an important constituent of human diet. DFs do affect our nutritional biochemistry and hence our health and well-being.” This may be common knowledge, but a reference is needed.

References for biological and food/digestion roles of polysaccharides have been added.

L55. P4 “Importantly for our current topic, it has been already established that the different local proton distributions for different spin label conformations can lead to delay-timedependent variations of the electron spin-spin distance distributions even in well-structured biopolymers.”

I don’t understand what you mean. Is this what you mean?

“Importantly, it has been already established that different spin label conformations, and their associated local proton concentration, around can affect the outcome electron spin-spin distance measurements.”

We rewrote this section.

L33 p5, “These interactions include electrostatic contributions and hydrophobic effects, they depend on steric features of particular chains and small molecules, as well as on the hydration of the specific moieties. Such interactions affect the conformational distributions, and also determine the propensity for agglomeration, gelation or liquid-liquid phase separation (LLPS) phenomena”

Do you mean “These interactions, e.g. electrostatic, hydrophobic effects, or hydration, depend on the steric features of the chains and small molecules. They affect the conformational distributions, and determine the propensity to agglomerate, form gels or make liquid-liquid phase separation (LLPS) phenomena

We have rewritten this section, using similar wording as you suggested..

L49 p5 “This characteristic of DFs is important in relation to their role in human nutrition.”

Can you add a ref?

The introduction of the previous version had two sections on dietary fibers, which were interspersed with information on EPR spectroscopy. We have rewritten this section so that we only have a single introductory section on DFs.

L11 p6 In ref 46, my recollection is that the protons on the biradical are important, is RIDME sensitive to this? The first author on ref 46 has a recent publication in JPCL that somewhat contradicts the point developed.

Overall, I doubt RIDME is helpful for biradical design but, it this is important, your point is actually very relevant when one wants to understand if a biradical has an affinity for a surface in DNP. I think you should consider this instead.

Essentially, the ih-RIDME experiment is sensitive to the proton spins beyond the spectral diffusion barrier, regardless whether they belong to the same molecule or not. The primary polarization transfer away from the paramagnetic centers is the most difficult to investigate step, due to the lack of spectroscopic techniques. We mean that ih-RIDME can be very useful for this specific task. We introduced the following wording, to clarify this a bit better: “the nearest protons that are placed just beyond the spectral diffusion barrier are of major importance for the primary transfer of the polarization away from the paramagnetic center.”

L31 p6. “quite well-know” is unnecessary, it’s well-known within the EPR community only. “based on a the relaxation-induced dipolar modulation enhancement pulse EPR technique (RIDME). The technique was originally designed to measure electron spinspin interactions,48,49 as a possible alternative to the commonly used double electron-electron resonance (DEER) technique.”

We now use similar wording as you suggested.

L41 p6. “can be quite reliably and accurately” isn’t it the same?

This paragraph was going into too much detail for an introductory paragraph. Given that the Introduction was too long, we shortened it considerable, which eliminated this phrase.

L30, p7. “At the same time, RIDME was found to characterise the structure of the local protonic environment of a spin label.” This redundant

This sentence is removed.

L23, p8. Typo at “deviation”

The typo is corrected.

L37, p8 “In this work we always recorded five traces, and an additional reference trace with short mixing time.” This is not five, but six traces.

This is rephrased to: “In this work, we always recorded five main traces, and an additional sixth reference trace with short mixing time.”

L5, p9, where does D/sigma^3 appears in the equation? Please clarify.

We are afraid that we cannot go into too much detail of the theory derived in reference 47 (ref. 53 in the second revised version). To improve clarity, we added that D/sigma^3 allows for normalization of the dipolar frequency correlation function.

L55, p9, “of course” is not needed.

Corrected.

L49, p10. “In particular, there are indications that the width of the component appearing at the highest local proton concentration might be unstable” Do you mean “In particular, it seems that highest local concentration distribution is unstable”?

No, we don’t mean the whole distribution – just its peak at highest concentrations, and, more specifically, only its width, but not the mean value. We slightly changed wording to make this more clear.

L29, p11 “Overall, these experiments indicate that the ih-RIDME data fitting can help following the interchain contacts statistics with good resolution. In our opinion, this is a very valuable result, as such contacts statistics is one of the key questions to ask when one wants to describe weak aggregates, including LLPS phenomena in biopolymers studies.”

Do you mean “Overall, these experiments demonstrate that the ih-RIDME data fitting can track interchain contact statistics with high resolution. Us understanding such contact statistics is crucial for characterizing weak aggregates, including phenomena like LLPS, in biopolymer studies, making this outcome is highly valuable”.

We accepted this suggestion. The text is modified accordingly.

L47, p13 “which would go far beyond the scope” may be “which would go beyond the scope”

Accepted.

L18, p20 “We anticipate that ih-RIDME and hf-DEER techniques might attract significant attention in the DNP research, because these techniques aim precisely at filling this gap of information on the local proton distributions around paramagnetic centers.”

I think you have a point, RIDME could help understand radical affinity with sample. This is something that would be good for DNP. When it comes to understand radicals, I could oppose the following arguments:

- can the ZQ e-e cross-relaxation contaminate the RIDME decay? If yes, how do you remove it?
- In DNP glass forming solution, would we see a difference between biradicals? And what would you understand from it? In general, biradicals are made to be as soluble as possible to avoid aggregation. To probe aggregation, a DEER decay would prove more useful?
- The ENDOR spectra may be in general more useful as Ref 46 shows protons with large hyperfine couplings are important.
- Can MIMS ENDOR be helpful in that case, the intensity of the central ENDOR peak correlates with local proton concentration?

We did not yet systematically test the performance of ih-RIDME in biradical samples and therefore we have decided to shorten the discussion of the relevance for DNP considerably instead of going into more detail on this issue. Although we did not add this to the manuscript, we would like to emphasize that ENDOR and ih-RIDME are designed to target different types of protons: the protons in the space within the spectral diffusion barrier and the protons in the space beyond this barrier, accordingly.
